# The role of melatonin in amyloid beta-induced inflammation mediated by inflammasome signaling in neuronal cell lines

**DOI:** 10.1038/s41598-023-45220-1

**Published:** 2023-10-19

**Authors:** Chutikorn Nopparat, Anuttree Boontor, Suchanoot Kutpruek, Piyarat Govitrapong

**Affiliations:** 1 Innovative Learning Center, Srinakharinwirot University, Sukhumvit 23, Bangkok, 10110 Thailand; 2 Chulabhorn Graduate Institute, Chulabhorn Royal Academy, Laksi, Bangkok, Thailand

**Keywords:** Neuroscience, Cellular neuroscience

## Abstract

Alzheimer's disease (AD) is the most prevalent neurodegenerative disorder. In addition to amyloid beta (Aβ) and tau, neuroinflammation is a crucial element in the etiology of this disease. However, the relevance of inflammasome-induced pyroptosis to AD is unknown. We aimed to clarify whether the anti-inflammatory effects of melatonin could prevent Aβ-mediated activation of the inflammasome. We demonstrated that Aβ upregulated NOD-like receptor family pyrin domain-containing 3 (NLRP3), apoptosis-associated speck-like protein containing a CARD, and cysteinyl aspartate-specific proteinase caspase (caspase 1) expression in SH-SY5Y neuroblastoma cells, resulting in the release of proinflammatory cytokines, including interleukin-1β (IL-1β), interleukin-18 (IL-18) and tumor necrosis factor (TNF-α). Melatonin prevented inflammasome signaling and excessive cytokine release caused by Aβ. We found that ethyl 2[(2-chlorophenyl)(hydroxy) methyl]acrylate (INF-4E, NLRP3 and caspase 1 inhibitor) significantly abolished Aβ-induced proinflammatory cytokine expression. The increase in cleaved-caspase 1, pro-IL18, and cleaved-IL18 caused by Aβ suggested the occurrence of pyroptosis, which was further confirmed by the increased expression of N-terminal gasdermin D (N-GSDMD). Melatonin plays a protective role against Aβ-induced inflammation via an inflammasome-associated mechanism that is essential in inducing the active forms of cytokines and pyroptosis. The ability of melatonin to inhibit inflammasome may represent a turning point in the treatment of AD progression.

## Introduction

Alzheimer's disease (AD) is the most prevalent neurodegenerative disorder associated with aging. Its symptoms include sustained neuroinflammation, inevitable memory loss, and cognitive deterioration^1^. Despite enormous efforts in Alzheimer's research, there is currently no viable treatment to prevent this progressive disease. Recent evidence has demonstrated that anti-inflammatory agents may play essential roles in AD pathogenesis^2^. In addition to amyloid (Aβ) and tau, which are the classic pathological hallmarks of AD, there is a substantial body of evidence demonstrating the involvement of neuroinflammatory cytokines and inflammasomes in Alzheimer's disease^3^.

Inflammation is one of the crucial mechanisms that induces tissue damage if the levels of inflammation exceed physiological levels. Neuroinflammation is the inflammatory response in the CNS that occurs in response to infection, trauma, ischemia, or toxin agents. In some cases, neuroinflammation can cause cell death through apoptosis or induce many neurodegenerative diseases^4^. Inflammasomes are closely involved in the neuroinflammation pathway. Inflammasomes are intracellular proinflammatory pattern recognition receptors (PRRs) that promote inflammation by producing proinflammatory cytokines^5^. Inflammasomes are stimulated by a number of factors, including reactive oxygen species (ROS), microbial or damage-associated stimuli, aggregated proteins, and metabolic disturbances. Inflammasomes are large multiprotein complexes that first recruit proinflammatory cysteinyl aspartate-specific proteinases (caspases) through apoptosis-associated speck-like protein containing a CARD (ASC) and subsequently cleave the precursors of proinflammatory cytokines to generate the mature forms of these cytokines^6^. ASC is mobilized during inflammasome assembly to create the ASC speck, which is a large, solitary paranuclear structure required for the recruitment of caspase 1 and its inflammatory effects^7^. In particular, the NLR family pyrin domain-containing 3 (NLRP3) inflammasome is significantly associated with inflammatory reactions. Activation of the NLRP3 inflammasome requires two steps. First, microbial chemicals or endogenous cytokines activate the nuclear factor kappa B (NF-κB) pathway, which increases the generation of NLRP3 and cytokines such as interleukin-1 (IL-1)^8,9^. Second, active caspase 1 in the inflammasome can activate and increase the signaling of cytokines, including interleukin-1β (IL-1β), interleukin-18 (IL-18) and tumor necrosis factor (TNF-α)^10,11^.

It is widely acknowledged that Alzheimer's disease is characterized by aberrant neuroinflammation caused by amyloid beta (Aβ)^1^, which results in memory impairment. Aβ has been shown to stimulate the NLRP3 inflammasome to promote IL-1β and neuroinflammation, thus contributing to the progression of AD^2^. Autophagy-mediated reductions in Aβ plaque levels inhibit the NLRP3 inflammasome. Aβ oligomers have been shown to result in the interaction between NLRP3 and ASC^6,12^. Inflammasome assembly leads to the cleavage of many pro-forms of cytokines, and the release of IL-1β induces cell death. Understanding how inflammasomes induce neuroinflammation in AD may lead to a novel therapeutic strategy for this disease. Recently, it has been suggested that suppressing the activity of NLRP3 inflammasome has substantial potential to prevent and treat AD^13^. Consequently, while developing therapeutic alternatives for Alzheimer's disease based on the manipulation of cytokine signaling, it is critical to investigate whether feedback mechanisms in cytokine signaling affect the expected positive outcome.

Melatonin is a hormone produced by the pineal gland and mitochondria. Melatonin is commonly used in the treatment of a wide range of disorders due to the numerous biological effects it has, including anti-inflammatory, antioxidative, antiapoptotic, and immunomodulatory properties^14,15^. Melatonin consistently inhibits oxidative stress, decreases innate immune activation, and enhances mitochondrial function in acute and chronic inflammation and aging experiments^16^. Our group previously reported that melatonin inhibited inflammation via NF-κB and cytokine signaling cascades^17,18^. Moreover, melatonin attenuated the amyloidogenic pathway and Aβ production in an AD model^19,20^. However, the role of melatonin in suppressing activation of the NLRP3 inflammasome in Alzheimer's disease is not well understood.

Herein, we aimed to examine the protective effects of melatonin against amyloid-beta-induced neuroinflammation through inflammasome signaling activation.

## Materials and methods

### Chemicals and reagents

The chemicals used in this study were purchased from the following sources: minimum essential medium (MEM), Ham’s F-12 medium, fetal bovine serum (FBS), penicillin and streptomycin were obtained from Gibco BRL (Gaithersburg MD, USA) and melatonin and luzindole were obtained from Sigma-Aldrich (St Louis, MO, USA). Human dopaminergic neuroblastoma SH-SY5Y cell lines were obtained from the American Type Culture Collection (Manassas, VA, USA). Corning culture plates and flasks were obtained from Corning Incorporated (Acton, MA, USA). ECL Prime Western Blotting Reagent® was purchased from GE Healthcare (Little Chalfont, Buckinghamshire, UK). All other chemicals used in this study were analytical grade and were obtained from Sigma Aldrich (St Louis, MO, USA) or Lab-scan analytical science (Dublin, Ireland). INF-4E was obtained from Torcris Bioscience (UK). Aβ42 was purchased from Anaspec (Fremont, CA, USA). All antibodies used in this study are listed in Table 1.

**Table 1:** Lists of antibodies.

Antibodies	Catalogue no	Dilution	Sources
Primary antibodies			
Rabbit anti-NLRP3	PA1665	1:1000	Boster Biological Technology, USA
Rabbit anti-ASC	DF6304	1:1000	Affinity Biosciences, USA
Rabbit anti-Caspase 1	AF5418	1:1000	Affinity Biosciences, USA
Mouse anti-IL-18	MABF2674	1:500	EMD Millipore Corporation, USA
Mouse anti IL-1β	#12242	1:1000	Cell Signaling Technology, Inc., USA
Rabbit anti-TNF-α	#3707	1:1000	Cell Signaling Technology, Inc., USA
Rabbit anti-cleaved Gasdermin D	#36425	1:1000	Cell Signaling Technology, Inc., USA
Mouse anti-Actin	MAP1501	1:20,000	EMD Millipore Corporation, USA
Secondary antibodies (peroxidase conjugated)			
Goat anti-Mouse IgG	AP124P		Merck Millipore, USA
Goat anti-Rabbit IgG	AP132P		EMD Millipore Corporation, USA

### Solubility of Aβ42 and melatonin

#### Preparation of amyloid beta peptide

The amyloid beta peptide was meticulously prepared following a previously established protocol^21^. A lyophilized white powder of 5 mg Aβ42 was reconstituted in 1% NH_4_OH, diluted in PBS, and stored at − 80 °C as the stock solution. The working solution, containing 100 µM aggregated Aβ42, was obtained by diluting the stock solution in serum-free growth media without penicillin/streptomycin and incubating it for 5 days at 37 °C. Dilutions were expertly crafted to achieve the desired concentrations using growth media supplemented with 1% FBS.

Melatonin was freshly prepared^20^ before each experiment as a 10 mM stock solution (in 40% EtOH) from which appropriated serial dilutions were mixed up (0.0004% EtOH for melatonin-treated and untreated controls).

#### Cell culture and treatment

Human dopaminergic neuroblastoma SH-SY5Y cells were cultured at 37 °C in 5% CO_2_ and 95% humidified air in minimum essential medium (MEM) supplemented with 45% Ham’s F-12, 10% inactivated fetal bovine serum (FBS), and 100 U/mL penicillin/streptomycin. SH-SY5Y cells were seeded in 60-mm Petri dishes and allowed to grow until they reached 80% confluence. To examine the effect of different concentrations of melatonin or Aβ, cells were then treated with and without various concentrations of melatonin (1 and 10 µM) or Aβ (0.1, 1, and 2 µM) in media containing 1% FBS for 24 h. After the optimal concentrations of melatonin and Aβ were determined, the cells were treated with 1 µM Aβ with and without 10 µM melatonin or were pretreated with 10 µM luzindole, a melatonin receptor antagonist, prior to 1 h of 10 µM melatonin treatment. Another experiment, cells were pretreated with 10 µM INF-4E, an inflammasome inhibitor, prior to 1 h of 10 µM melatonin treatment.

#### Western blot analysis

After the cultured cell were collected, RIPA lysis buffer was used to lyse the cells. The cell lysate was sonicated and centrifuged. The protein concentrations of the supernatants were quantified using a Bradford protein assay (Bonjoch and Tamayo, 2001). Protein samples were loaded onto SDS-PAGE gels to fractionate the target proteins and were transferred onto PVDF membranes. The PVDF membranes were blocked with 3% bovine serum albumin or 5% nonfat milk and then incubated with primary antibodies (Table 1). Bound primary antibodies were then detected by incubation with horseradish peroxidase (HRP)-conjugated anti-rabbit or anti-mouse secondary antibodies. Signals on the membrane were developed with chemiluminescent ECL reagent and detected by gel documentation. Band density quantification was performed by densitometry analysis by ImageJ (NIH, Bethesda, MD, USA), and the data were normalized using β-actin as an internal standard.

#### Immunocytochemistry

SH-SY5Y cells were incubated for 24 h in a 24-well plate containing complete medium and a coverslip that was precoated with poly-l-ornithine. The cells were then incubated with 10 µM melatonin for 2 h before 1 µM Aβ treatment for another 24 h. The cells were fixed in 4% paraformaldehyde in 0.1 M phosphate-buffered saline (PBS) for 20 min at room temperature, washed with PBS 3 times and blocked. Then, the cells were incubated with NLRP3 primary antibodies (1:200) at 4 °C overnight, incubated with Alexa 596-conjugated anti-rabbit (1:400) secondary antibodies, washed with PBS and mounted with antifade reagent (Vectashield, Vector Laboratories, Burlingame, CA) before being visualized under a microscope. NLRP3 staining was examined with a confocal laser scanning microscope (FV 3000, Olympus, Tokyo, Japan).

#### Alpha-LISA immunoassay

SH-SY5Y cells were seeded in 60-mm Petri dishes and allowed to grow until they reached 80% confluence. The cells were then incubated with 10 µM melatonin for 2 h followed by 1 µM Aβ treatment for another 24 h in a 95% humidified air incubator at 37 °C and 5% CO_2_. The levels of cytokines were detected according to the manufacturer’s protocol. Briefly, after the cells were collected, the cells were diluted in 0.6 mL of 10× Alpha LISA Immunoassay buffer, and then antibodies against different cytokine (IL-1β, IL-18 and TNF-α) were added, followed by the addition of the analysis solution in AlphaLISA Immunoassay buffer, which was then slightly vortexed with AlphaLISA Acceptor beads and mixed with 10 µL of Acceptor beads in AlphaLISA Immunoassay buffer. Alpha Donor beads were added, mixed thoroughly and then incubated in the dark at 23 °C for 60 min. Cell solutions were read by using an Alpha-capable instrument (EnVision® or EnSpire® Multilabel plate readers, USA).

### Statistical analysis

The data are expressed as the mean ± SEM. Statistically significant differences between the experimental group and the control group were determined by one-way analysis of variance (one-way ANOVA) and Tukey’s post hoc test using Graph Pad Prism version 7.0.4 (Graph Pad Software Inc., La Jolla, CA), and a *p* value < 0.05 was considered to be a statistically significant difference between the experimental group—and the control group.

## Results

### Effect of Aβ on inflammasome and inflammatory cytokine expression

**Figure 1:**
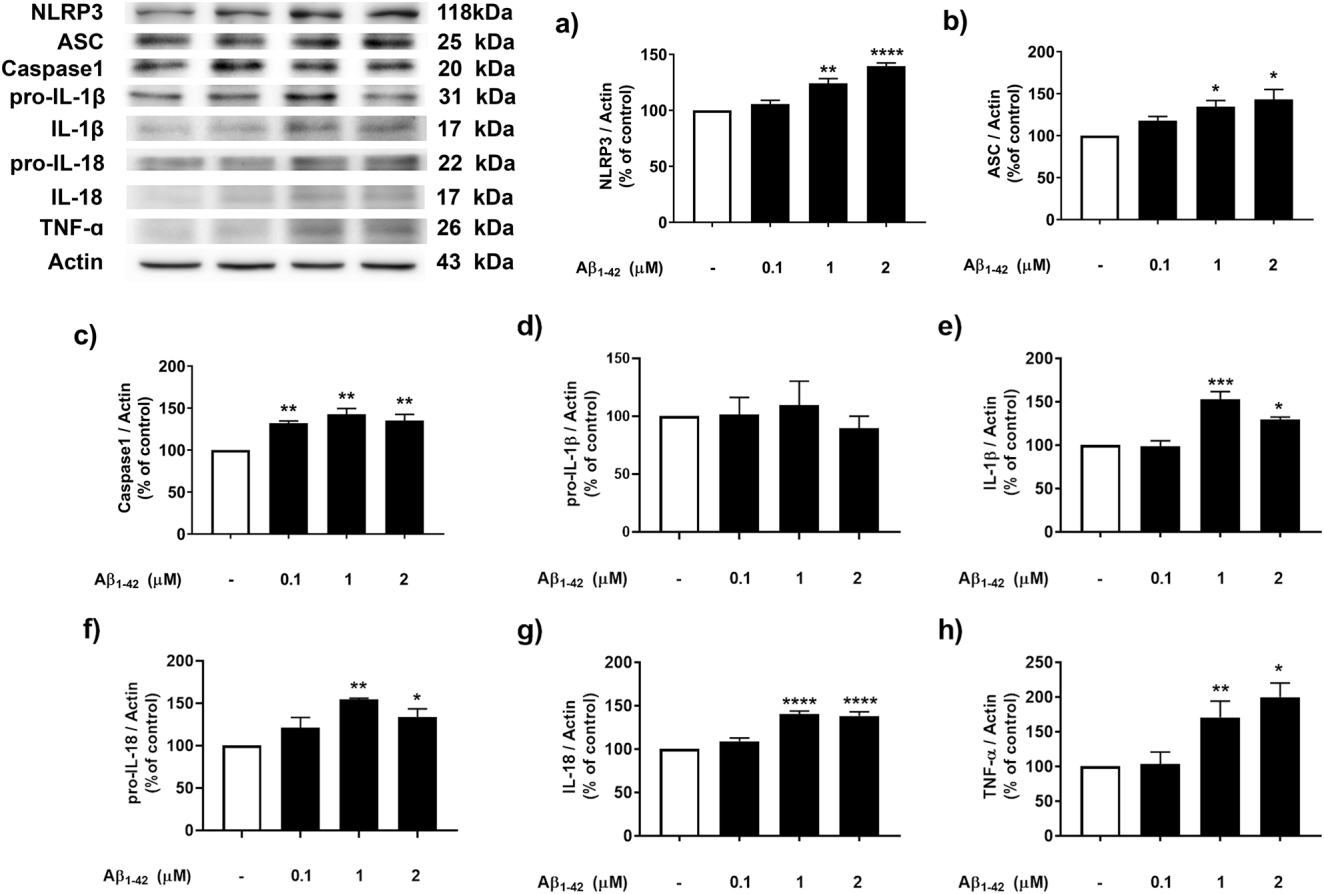
The concentration-dependent effect of Aβ on inflammasome protein and proinflammatory cytokine expression in SH-SY5Y cells. Cells were incubated with various concentration (01, 1 and 2 µM) of Aβ for 24 min. Western blot analysis was used to determine the expression levels of (**a**) NLRP3, (**b**) ASC, (**c**) Caspase 1, (**d**) pro-IL-1β, (**e**) IL-1β, (**f**) pro-IL-18, (**g**) IL-18 and (**h**) TNF-α. The band densities were normalized to actin. The ratios were calculated as a percentage of the respective value in the control group. The data are expressed as the means ± S.E.M. One-way ANOVA and Tukey's pos-hoc test were performed for statistical analysis. N = 3–4 (*, **, ***, ****denote statistical significance at *p* < 0.05, *p* < 0.01, *p* < 0.001 and *p* < 0.0001 compared to the control group, respectively).

To determine whether Aβ could affect inflammasome expression, SH-SY5Y cells were treated with various concentrations (0.1, 1, and 2 µM) of Aβ to determine the optimal toxic concentration to be used in the following experiments. The results showed that 1 and 2 µM Aβ significantly enhanced the expression of NLRP3 (*p* < 0.01, 0.0001, Fig. 1a), ASC (*p* < 0.05, 0.05, Fig. 1b) and caspase 1 (*p* < 0.01, 0.01, 0.01, Fig. 1c) compared with that in the control group (Fig. 1). These results indicated that 1 µM Aβ was the lowest concentration that induced inflammasome protein expression. To determine whether Aβ could induce the production of different proinflammatory cytokines, which are the downstream signaling targets of inflammasomes, SH-SY5Y cells were treated with various concentrations (0.1, 1, and 2 µM) of Aβ. The results showed that 1 and 2 µM Aβ did not alter the expression of pro-IL-1β (Fig. 1d), while 1 and 2 µM Aβ significantly enhanced the expression of IL-1β (*p* < 0.001, 0.05, Fig. 1e), pro-IL-18 (*p* < 0.01, 0.05, Fig. 1f), IL-18 (*p* < 0.0001, 0.0001, Fig. 1g) and TNF-α (*p* < 0.01, 0.05, Fig. 1h). Therefore, 1 µM Aβ was the optimal concentration for all subsequent experiments.

### Effect of melatonin on Aβ-induced inflammasome and proinflammatory cytokine expression

Various concentrations of melatonin were previously used by several investigators, such as, Fan and his team^22^ used 10 µM of melatonin to study autophagy and inflammasome activity in SH-SY5Y cell, and Waseem et al.^23^ showed that 10 µM melatonin reduced oxidative stress and apoptosis in SH-SY5Y cells. The concentrations of melatonin used in the present study were modified from various reports and one of our previous studies^21^.

**Figure 2:**
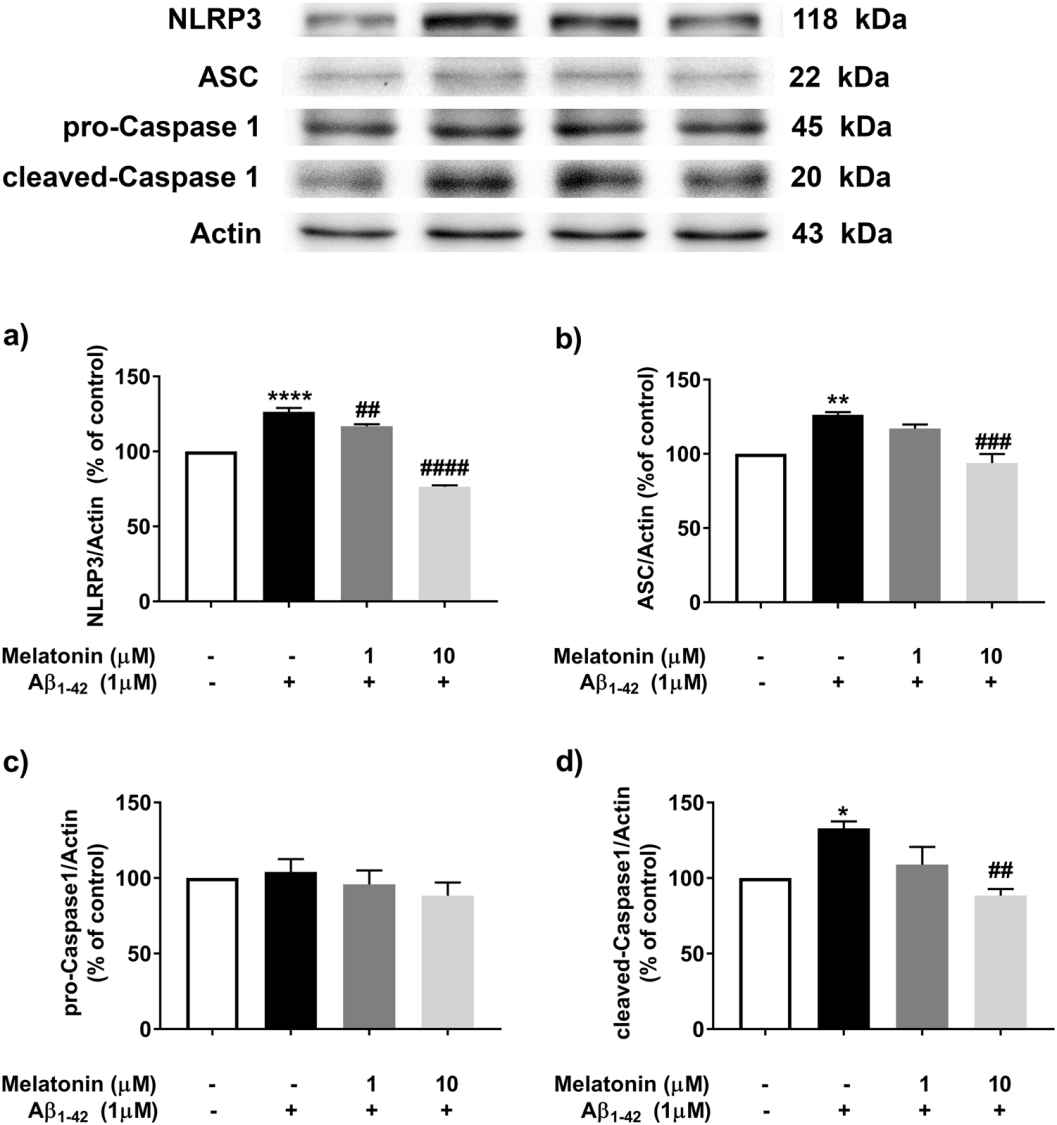
The concentration-dependent effect of melatonin on Aβ-induced inflammasome proteins expression in SH-SY5Y cells. Cells were pretreated with 1 µM or 10 µM melatonin for 2 h followed by 1 µM Aβ treatment. Western blot analysis was used to determine the expression levels of (**a**) inflammasome proteins including NLRP3, (**b**) ASC, (**c**) pro-Caspase 1 and (**d**) cleaved-caspase 1. The band densities were normalized to actin. The ratios were calculated as a percentage of the respective value in the control group. The data are expressed as the means ± S.E.M. one-way ANOVA and Tukey's pos-hoc test were performed for statistical analysis. N = 3–4 (*, **, ****denote statistical significance at *p* < 0.05, *p* < 0.01, and *p* < 0.0001 compared to the control group, and ^##^, ^###^, ^####^denote statistical significance at *p* < 0.01, *p* < 0.001, and *p* < 0.0001 compared to the Aβ treatment group, respectively).

After treatment with 1 µM Aβ, 1 and 10 µM melatonin were added and further incubated for 24 h. The levels of inflammasome signaling proteins, including NLRP3, ASC and pro- and cleaved-caspase 1, were investigated. The results showed that the expression levels of NLRP3 (*p* < 0.0001, Fig. 2a), ASC (*p* < 0.001, Fig. 2b) and pro- (Fig. 2c) cleaved-caspase 1 (*p* < 0.01, Fig. 2d) in cells that were pretreated with 10 µM melatonin followed by 1 µM Aβ were significantly decreased compared with those in cells treated with 1 µM Aβ alone.

**Figure 3:**
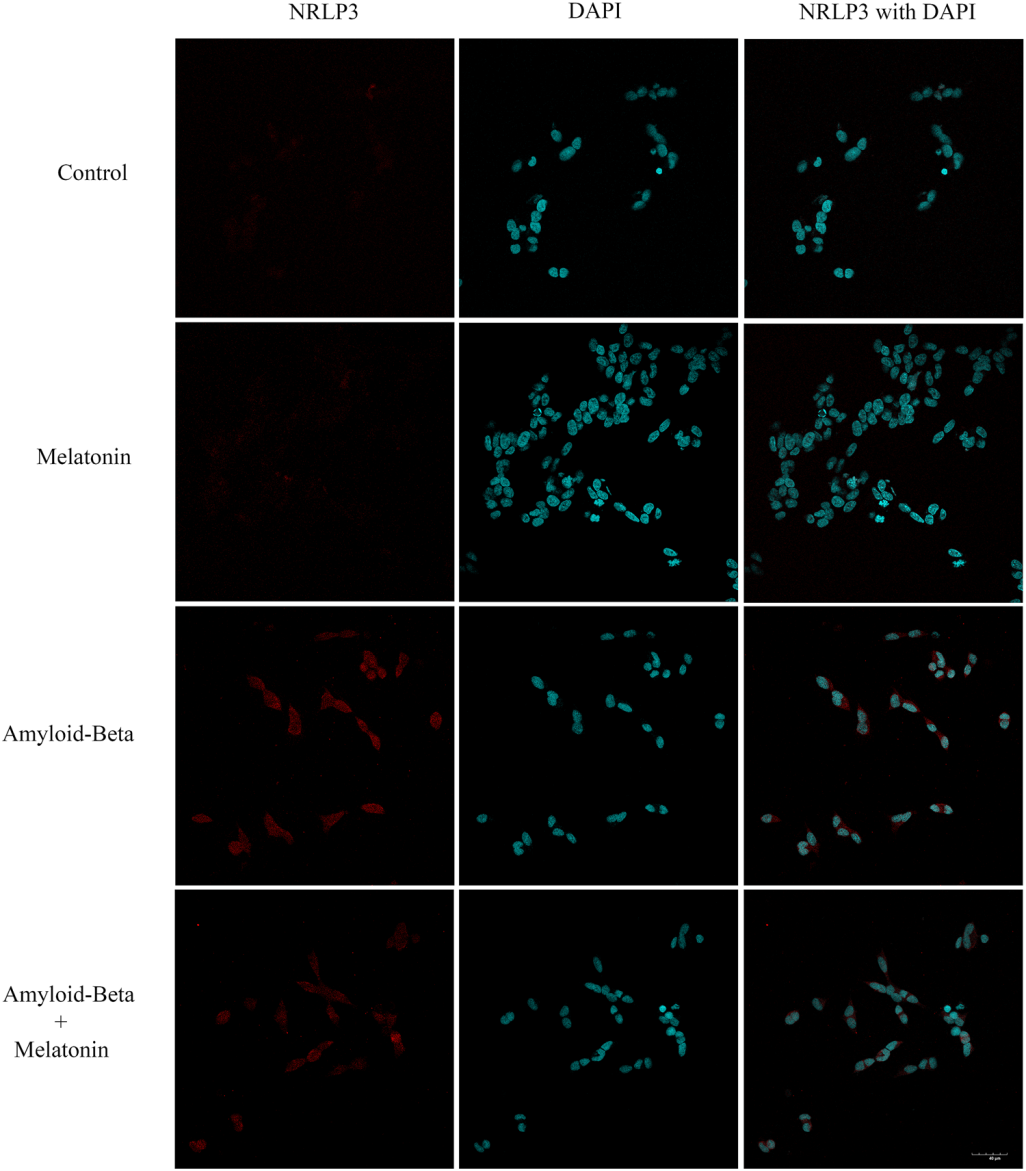
A representative image showing NLRP3 immunostaining in Aβ42 and melatonin-treated SH-SY5Y cell cultures. Control cells were incubated in serum free media for 24 h. Cells were treated with 1 µM melatonin for 24 h. Cells were treated with 1 µM Aβ42 for 24 h, and cells were pretreated with 1 µM melatonin for 2 h prior to 24 h of 1 µM Aβ42 treatment. The red color indicates NLRP3-positive immunostaining using Alexa 488-conjugated goat anti-rabbit IgG and DAPI nuclear staining (1:2000, blue color) on cover slips. The scale bar equals 30 μm.

The effect of Aβ and melatonin on the levels of NLRP3, which is the key inflammasome protein, was further assessed by immunohistochemical analysis (Fig. 3). The results showed that 1 µM Aβ increased the intensity of the red color representing NLRP3 immunostaining, whereas pretreatment with 10 µM melatonin for 2 h followed by 1 µM Aβ reduced the intensity of the red color. Taken together, these results indicated that melatonin protected against inflammasome activation in response to Aβ treatment.

**Figure 4:**
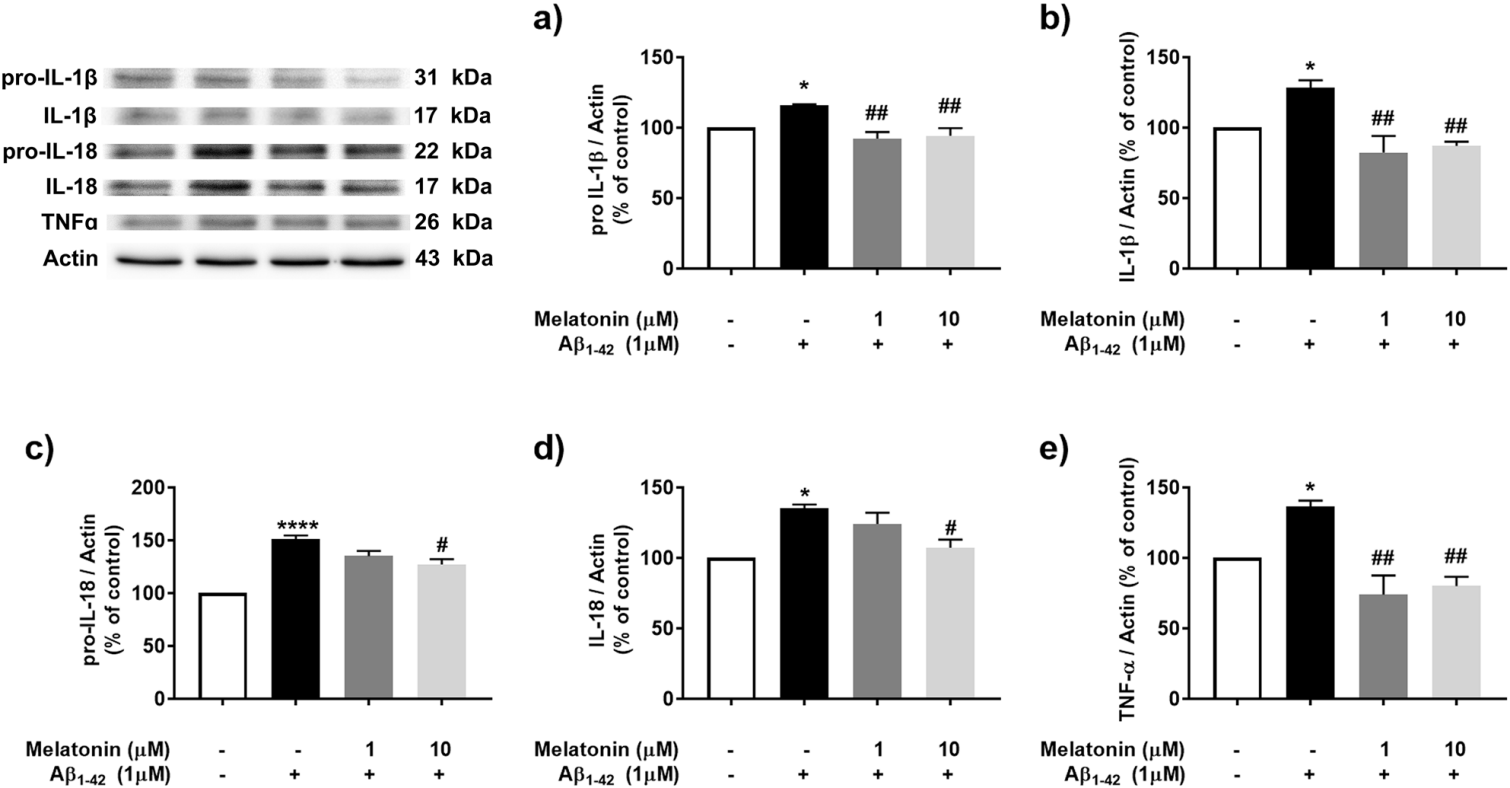
The concentration-dependent effect of melatonin on Aβ-induced cytokine expression in SH-SY5Y cells. Cells were pretreated with 1 µM or 10 µM melatonin for 2 h and followed by 1 µM Aβ treatment. Western blot analysis was used to determine the expression levels of cytokines including (**a**) pro-IL-1β, (**b**) IL-1β, (**c**) pro-IL-18, (**d**) IL-18 and (**e**) TNF-α. The band densities were normalized to actin. The ratios were calculated as a percentage of the respective value in the control group. The data are expressed as the means ± S.E.M. One-way ANOVA and Tukey's pos-hoc test were performed for statistical analysis. N = 3 (*, ****denote statistical significance at *p* < 0.05 and 0.0001 compared to the control group, and ^#^, ^##^denote statistical significance at *p* < 0.05 and *p* < 0.01, compared to the Aβ treatment group, respectively).

Moreover, several cytokines that are known as downstream signaling targets of inflammasomes, including IL-1β, IL-18 and TNF-α, were investigated after 1 µM Aβ treatment with or without pretreatment with 1 and 10 µM melatonin (Fig. 4). The results showed that 1 µM Aβ treatment significantly increased pro-IL-1β (*p* < 0.05, Fig. 4a), IL-1β (*p* < 0.05, Fig. 4b), pro-IL-18 (*p* < 0.0001, Fig. 4c) IL-18 (*p* < 0.05, Fig. 4d) and TNF-α (*p* < 0.05, Fig. 4e) expression compared with that in the control group, while pretreatment with 10 µM melatonin prior to 1 µM Aβ treatment significantly decreased pro-IL-1β (*p* < 0.01, Fig. 4a), IL-1β (*p* < 0.01, Fig. 4b), pro-IL-18 (*p* < 0.05, Fig. 4c) IL-18 (*p* < 0.05, Fig. 4d) and TNF-α (*p* < 0.01, Fig. 4e) expression compared with that in the Aβ treatment group (Fig. 4). These results indicated that 10 μM melatonin as an effective concentration for examining the effect of Aβ on the inflammasome signaling cascade.

**Figure 5:**
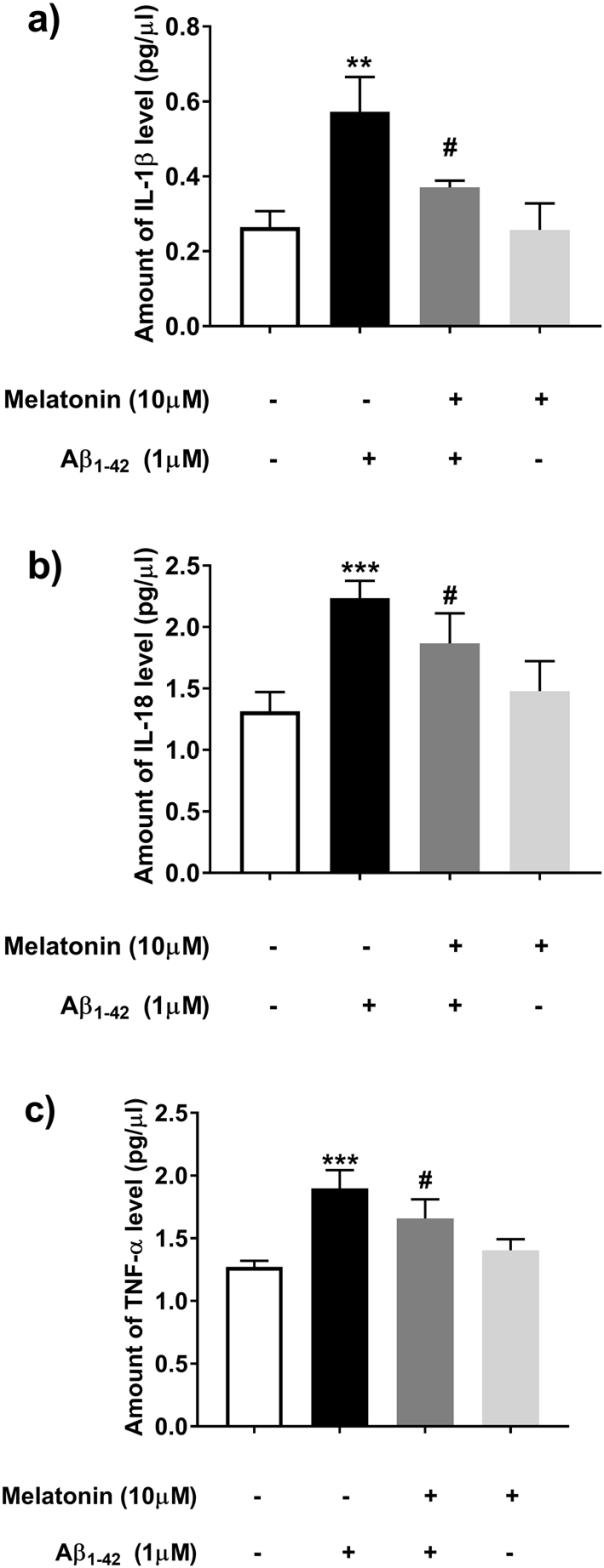
The effect of melatonin on Aβ-induced cytokine levels in SH-SY5Y cells. Cell were pretreated with 1 µM or 10 µM melatonin for 2 h followed by 1 µM Aβ treatment. Alpha-LISA analysis was used to determine the expression levels of cytokines including (**a**) IL-1β, (**b**) IL-18 and (**c**) TNF-α. Cell solutions were examined by using an Alpha-capable instrument. The values were calculated as the pg/µL of the standard value. The data are expressed as the means ± S.E.M. One-way ANOVA and Tukey's pos-hoc test were performed for statistical analysis. N = 4 (**, ***denote statistical significance at *p* < 0.01 and *p* < 0.001 compared to the control group, respectively, and ^#^denotes statistical significance at *p* < 0.05 compared to the Aβ treatment group).

We further tested the inflammatory reaction in response to Aβ-induced upregulation of various cytokines using AlphaLISA immunoassays. We found that pro-inflammatory cytokines [IL-1β (*p* < 0.01, Fig. 5a), IL-18 (*p* < 0.001, Fig. 5b) and TNF-α (*p* < 0.001, Fig. 5c)] were markedly increased following Aβ treatment, while pretreatment with 10 µM melatonin significantly inhibited Aβ-induced secretion of IL-1β (*p* < 0.05, Fig. 5a), IL-18 (*p* < 0.05, Fig. 5b) and TNF-α (*p* < 0.05, Fig. 5c).

### Protective effect of melatonin on the Aβ-induced pro-inflammatory cytokines via the melatonin Receptor

**Figure 6:**
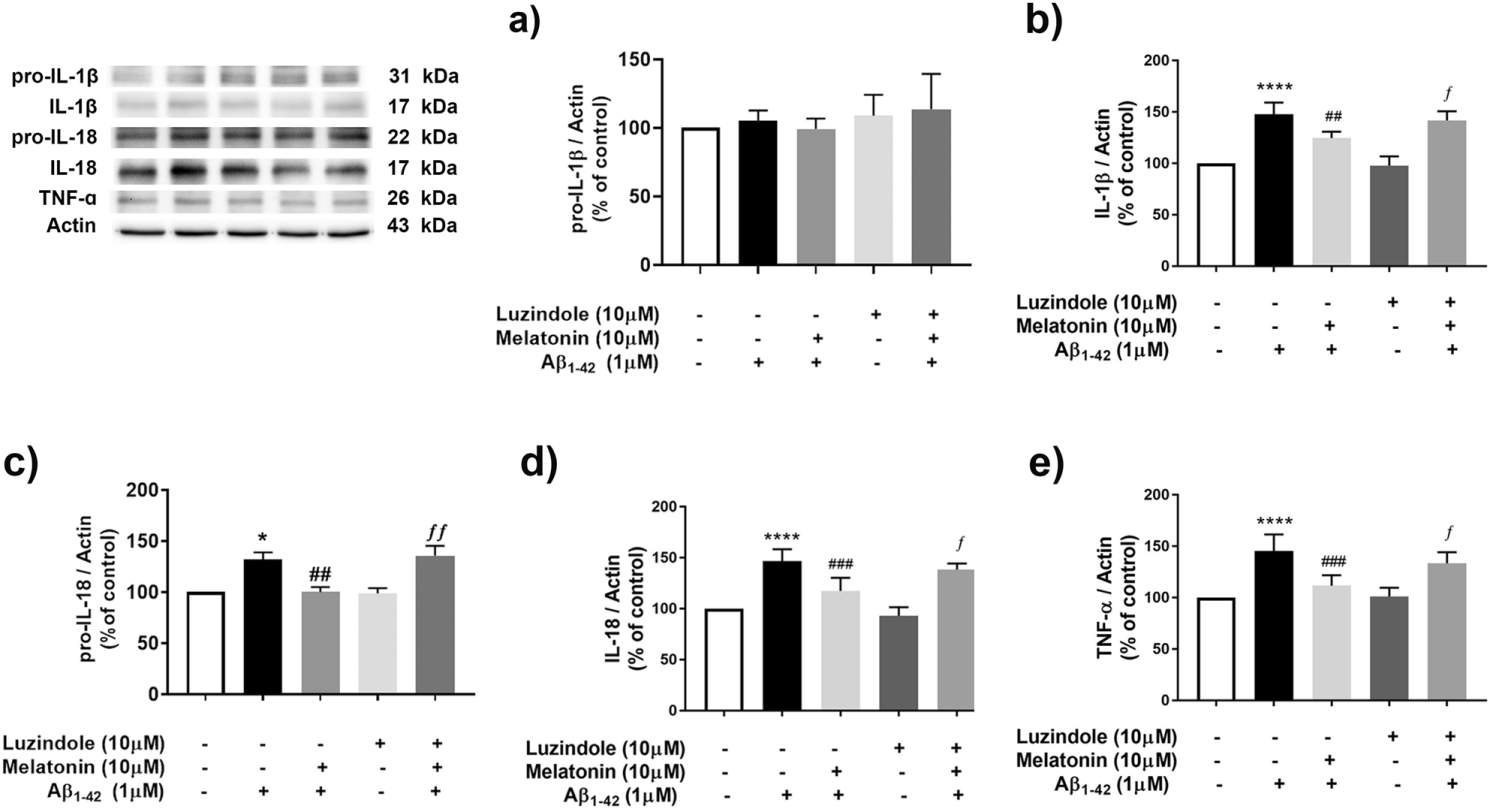
Protective effects of melatonin against Aβ-induced cytokines via the melatonin receptor in SH-SY5Y cells. Cells were treated with 1 µM luzindole 30 min prior to pretreatment with 10 µM melatonin for 2 h followed by 1 µM Aβ treatment. Western blot analysis was used to determine the expression levels of inflammatory cytokines including (**a**) pro-IL-1β, (**b**) IL-1β, (**c**) pro-IL-18, (**d**) IL-18 and (**e**) TNF-α. The band densities were normalized to actin. The ratios were calculated as a percentage of the respective value in the control group. The data are expressed as the means ± S.E.M. One-way ANOVA and Tukey's pos-hoc test were performed for statistical analysis. N = 4 (*, ****denote statistical significance at *p* < *0.05, p* < 0.0001 compared to the control group; ^##^, ^###^denote statistical significance at *p* < 0.01 and *p* < 0.001 compared to the Aβ treatment group, respectively; and ƒ and *ff* denote statistical significance at *p* < 0.05, 0.01 compared to melatonin pretreatment group).

To determine whether melatonin affects pro-inflammatory cytokines via the melatonin receptor during Aβ treatment, SH-SY5Y cells were treated with 10 µM luzindole, a melatonin receptor antagonist, 1 h prior to pretreatment with 10 µM melatonin for 2 h, followed by 1 µM Aβ treatment for another 24 h. and pro-inflammatory cytokines, including pro-IL-1β, pro-IL-18 IL-1β, IL-18 and TNF-α (Fig. 6), were measured. One micromolar Aβ treatment did not alter the expression of pro-IL-1 β (Fig. 6a), while it significantly increased the expression of IL-1β (*p* < 0.0001, Fig. 6b), pro-IL-18 (*p* < 0.05, Fig. 6c) IL-18 (*p* < 0.0001, Fig. 6d) and TNF-α (*p* < 0.0001, Fig. 6e) compared with that in the control group. Pretreatment with 10 µM melatonin for 2 h followed by 1 µM Aβ treatment significantly decreased the expression of IL-1β (*p* < 0.01, Fig. 6b), pro-IL-18 (*p* < 0.01, Fig. 6c) IL-18 (*p* < 0.001, Fig. 6d) and TNF-α (*p* < 0.001, Fig. 6e) compared with the response to 1 µM Aβ alone. Moreover, 1 µM luzindole significantly (*p* < 0.05, Fig. 6b,d,e and *p* < 0.01 for Fig. 6c) abolished the protective effect of melatonin against Aβ-induced proinflammatory cytokines (IL-1β, IL-18 and TNF-α) expression. These data indicated that the protective effect of melatonin against Aβ-induced cytokine expression acted via melatonin receptors.

### Amyloid beta-induced cytokine release via the inflammasome pathway

**Figure 7:**
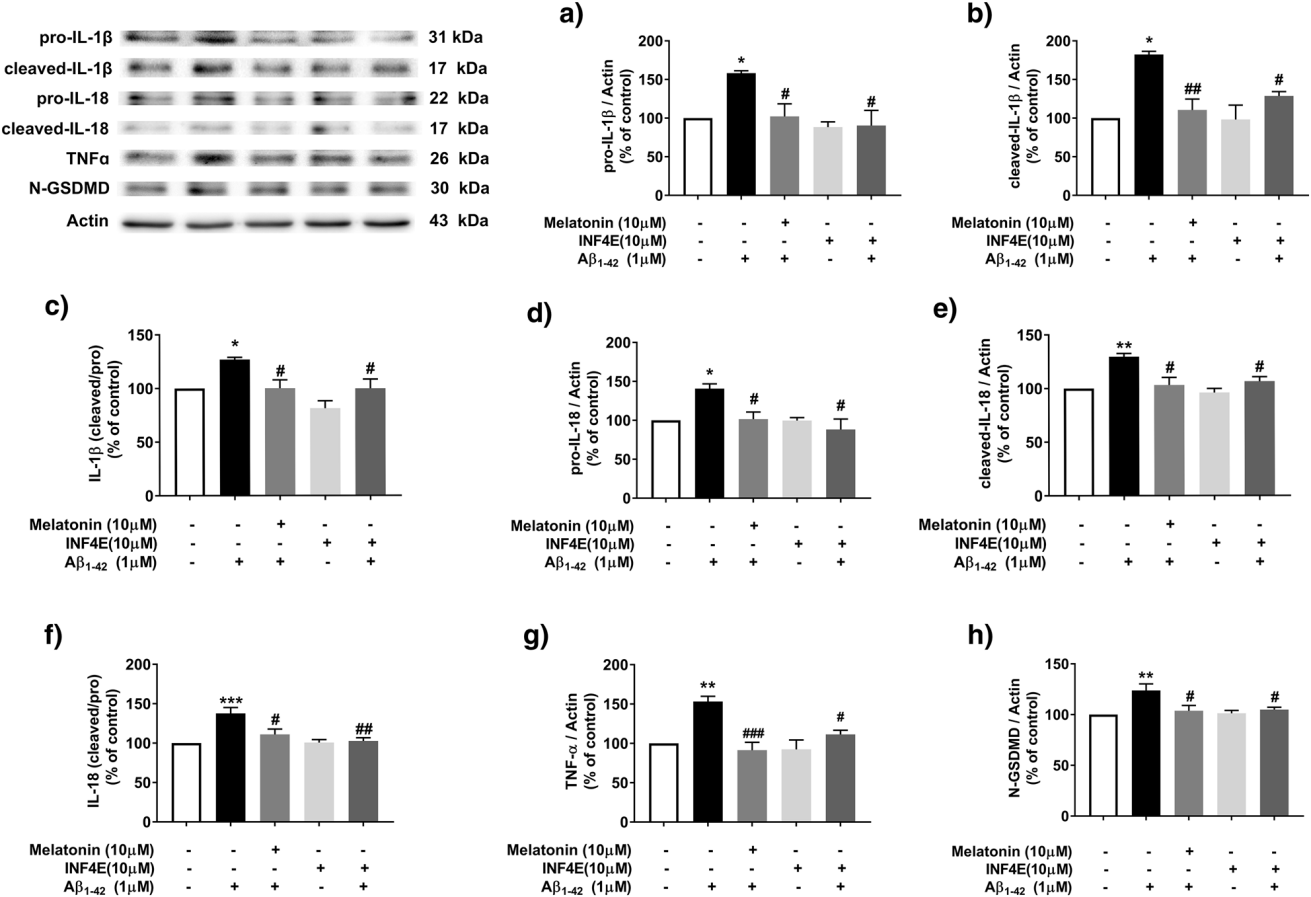
Effects of cytokines release via the inflammasome in Aβ-treated SH-SY5Y cell. Cell were treated with 1 µM INF-4E (NLRP3, caspase 1 inhibitor) 30 min prior to pretreatment with 10 µM melatonin for 2 h followed by 1 µM Aβ treatment. Western blot analysis was used to determine the expression levels of (**a**) pro-IL-1β, (**b**) cleaved-IL-1β, (**c**) cleaved/pro-IL-1β, (**d**) pro-IL-18, (**e**) cleaved-IL-18, (**f**) cleaved/pro-IL-18 and (**g**) TNF-α and (**h**) Gasdermin D. The ratios were calculated as a percentage of the respective value in the control group. The data are expressed as the means ± S.E.M. One-way ANOVA and Tukey's pos-hoc test were performed for statistical analysis. N = 4 (*, **, ****denote statistical significance at *p* < 0.05, *p* < 0.01, and *p* < 0.0001 compared to the control group and ^#^, ^##^, ^###^denote statistical significance at *p* < 0.05, *p* < 0.01 and *p* < 0.001 compared to the Aβ treatment group, respectively).

Previous results indicated that melatonin attenuated Aβ-induced inflammasome protein and downstream cytokine expression. **To determine whether** Aβ**-induced cytokine release was induced by inflammasome activation**, SH-SY5Y cells were treated with 1 µM INF-4E, an NLRP3 inflammasome and caspase 1 inhibitor, for 30 min followed by 1 µM Aβ treatment for another 24 h or pretreatment with 10 µM melatonin for 2 h followed by 1 µM Aβ treatment for another 24 h. Downstream inflammasome cytokines, including pro- and cleaved-IL-1β and IL-18 and TNF-α, were investigated (Fig. 7).

One micromolar Aβ treatment significantly increased the expression of the pro-IL-1β (*p* < 0.05, Fig. 7a), cleaved-IL-1β (*p* < 0.05, Fig. 7b), the ratio of cleaved/pro-IL-1β (*p* < 0.05, Fig. 7c), pro-IL-18 (*p* < 0.05, Fig. 7d), cleaved IL-18 (*p* < 0.01, Fig. 7e), the ratio of cleaved/pro-IL-18 (*p* < 0.01, Fig. 7f) and TNF-α levels (*p* < 0.01, Fig. 7g) compared with those in the control group. Pretreatment with 10 µM melatonin for 2 h followed by 1 µM Aβ treatment significantly decreased the expression of pro-IL-1β (*p* < 0.05, Fig. 7a), cleaved-IL-1β (*p* < 0.01, Fig. 7b), the ratio of cleaved/pro-IL-1β (*p* < 0.05, Fig. 7c), pro-IL-18 (*p* < 0.05, Fig. 7d), cleaved-IL-18 (*p* < 0.05, Fig. 7e), the ratio of cleaved/pro-IL-18 (*p* < 0.05, Fig. 7f) and TNF-α levels (*p* < 0.001, Fig. 7g) compared with 1 µM Aβ alone. Moreover, 1 µM INF-4E significantly (*p* < 0.05, Fig. 7a–g) abolished the effect of Aβ-induced all pro-inflammatory cytokine expression.

These results indicated that melatonin attenuated Aβ-induced NLRP3 pathway activation by inhibiting proteins related to the NLRP3 pathway and thereby suppressing the expression of IL-1β, IL-18 and TNF-α. The increase in cleaved-caspase 1, pro-IL18 and cleaved-IL18 caused by Aβ suggested the occurrence of pyroptosis. To further confirm the presence of pyroptosis under the same treatment conditions, 1 µM Aβ was administered and significantly increased the expression of N-terminal gasdermin D (N-GSDMD) (*p* < 0.01, Fig. 7h), an effector of pyroptosis that is downstream of the inflammasome signaling pathways, compared with that in the control group, whereas pretreatment with 1 µM INF-4E for 30 min or pretreatment with 10 µM melatonin for 2 h followed by 1 µM Aβ treatment significantly decreased levels of N-GSDMD (*p* < 0.05, Fig. 7h) compared with Aβ alone. These data indicated that the protective effect of melatonin against Aβ-induced inflammation via the inflammasome pathway was essential for inducing the active forms of cytokines and the pyroptosis signaling pathway, and these effects were significantly reversed by melatonin (Fig. 7h).

## Discussion

The two main findings from this study of Aβ-treated neuronal cells are as follows: (1) Aβ-treated neuronal cells exhibited increased neuroinflammation by promoting inflammasome expression, and (2) melatonin inhibited inflammation and pyroptosis mediated by the NLRP3 signaling pathway.

A previous study indicated that inflammation was one of the genetic and environmental risk factors that induced AD progression^24^. Activation of NF-κB can induce downstream signaling cytokines such as IL-1β, IL-6 and TNF-α^25,26^. The NF-κB pathway is essential for cytokine activation and AD pathology. Conversely, Aβ aggregation induces the NF-κB pathway and inflammasome activation^3^, which can act as a vicious cycle to increase AD progression. The unnatural accumulation of Aβ in the brain is an early characteristic of AD that is typically accompanied by neuronal loss and inflammatory responses. Many previous works have indicated that Aβ can induce NLRP3 inflammasome activation in microglia^3,27^.

The present study in neuronal cell lines demonstrated that 1 µM Aβ could upregulate NLRP3, the key component of the inflammasome. NLRP3, ASC and Caspase 1 expression was induced by Aβ treatment. The present data are compatible with recent studies which showed that Aβ exposed to SH-SY5Y cells caused NLRP3 inflammasome activation^28,29^.

Our data also showed that during the last step of the inflammasome process, inactive pro-caspase 1 was cleaved to form active caspase 1, which activates many proinflammatory cytokines^30^. In addition, AD mice exhibited NLRP1 and NLRP3 upregulation and IL-1β and IL-18 production, which further contributed to the onset of AD^31^. Inhibition of NLRP3 activation has been suggested as an emerging therapeutic approach in the management and treatment of AD. Therefore, the effect of the NLRP3 inflammasome is an effective therapeutic target for AD.

The role of melatonin in reducing inflammation is supported by a number of lines of evidence. For example, melatonin attenuates NF-κB and many inflammatory cytokines in aging mice^18^. Melatonin can protect against H_2_O_2_-induced inflammation and cell cycle arrest in SH-SY5Y cells^17^. Melatonin-mediated inhibition of inflammasome complex formation has been reported^32^. In the present study, pretreatment with 1 µM and 10 µM melatonin attenuated the expression of all inflammasome components, including ASC, NLRP1 and active caspase 1. In addition, proinflammatory cytokines, including IL-1β, IL-18 and TNF-α, were decreased after pretreatment with 10 µM melatonin.

Typically, the mechanism of melatonin can be receptor dependent or independent. Melatonin can bind to melatonin receptor (MT) in the plasma membrane, some intracellular proteins and orphan nuclear receptors or exert potent antioxidant effects^33^. To determine the site at which melatonin inhibits the inflammasome, luzindole, a melatonin receptor antagonist, was used. Our results showed that melatonin could act via the receptor to regulate cytokines during Aβ treatment. However, the expression levels of inflammasome proteins were not different in response to pretreatment with melatonin followed by 1 µM Aβ treatment and then adding 1 µM luzindole. This finding suggested that the effect of melatonin on inflammasome attenuation did not involve the melatonin receptor. Melatonin has many targets by which to regulate cytokine expression, and it is possible that melatonin can act as an antioxidant to regulate inflammasomes but act on receptors to affect other inflammation pathways, such as the NF-κB pathway. Our previous reports showed that the protective effect of melatonin on methamphetamine-induced cytokine expression via the NF-κB signaling pathway was abrogated by luzindole or melatonin receptor knockdown^34^.

One interesting point associated with cytokine expression and activation is the activation of both the NF-κB cascade and inflammasome pathway^35^. Our previous study showed that Aβ_42_ significantly increased the relative levels of pNF-κB/ NF-κB and Aβ42-induced nuclear translocation of NF-κB65. Melatonin pretreatment significantly maintained the normal NF-κB expression^21^. One possible mechanism to explain the relationship between inflammasome activation and cytokines is the process by which active caspase 1 changes cytokines to an active form. When caspase 1 was inhibited by a caspase 1 inhibitor, the subsequent cleavage and release of active IL-1β were abrogated^36^. To determine whether melatonin affects Aβ-induced cytokine upregulation via the inflammasome pathway, INF-4E, an inflammasome inhibitor, was used. Since NF-κB is the main mechanism that induces full-length IL-1β (pro-form) expression and the inflammasome process cleaves pro-IL-1β to active IL-1β, we examined the ratio of the pro-form and active-form of IL-1β and IL-18 after the use of an inflammasome inhibitor to determine the exact pathway by which melatonin attenuates cytokine levels. The results showed that the effect of melatonin on Aβ-induced cytokine upregulation involved the inflammasome pathway. This result indicated that melatonin played an important role in cytokine activation via the inflammasome pathway and that this pathway might act as a vicious cycle to induce more Aβ accumulation in AD.

In addition, active IL-1β and IL-18 induced by inflammasome activation closely correlate with pyroptosis and cell death. Recent evidence suggests that pyroptosis due to NLRP3 activation induces AD^37^. The mechanisms of pyroptosis depend on NLRP3, caspase 1 and the GSDMD protein family. The activation of pro-caspase 1 results in the formation of active caspase 1, which cleaves gasdermin D (GSDMD) to liberate its N-terminal domain (N-GSDMD). The N-terminal domain then binds to phosphatidylinositol phosphates and phosphatidylserine in the cytomembrane; as a result, a lytic form of cell death known as pyroptosis is induced.

Our findings demonstrate the protective effect of melatonin against the expression of N-GSDMD, an important protein in the GSDMD family, via inflammasome signaling. This protein forms pores in the plasma membrane that lead to cell death. This result indicated that the crucial role of N-GSDMD was to facilitate pore formation in the cell membrane^37^, and inflammatory cytokines produced by inflammation and the inflammasome went through these pores to generate an inflammatory response and induce AD. Melatonin can ameliorate the inflammatory response not only via the NF-κB pathway^18^ but also regulate the inflammasome pathway by decreasing ASC and NLRP3 to activate caspase 1. The key roles of active caspase 1 involve the cleavage of IL-1β, IL-18 and N-GSDMD to induce pyroptotic cell death and excessive levels of cytokines.

**Figure 8:**
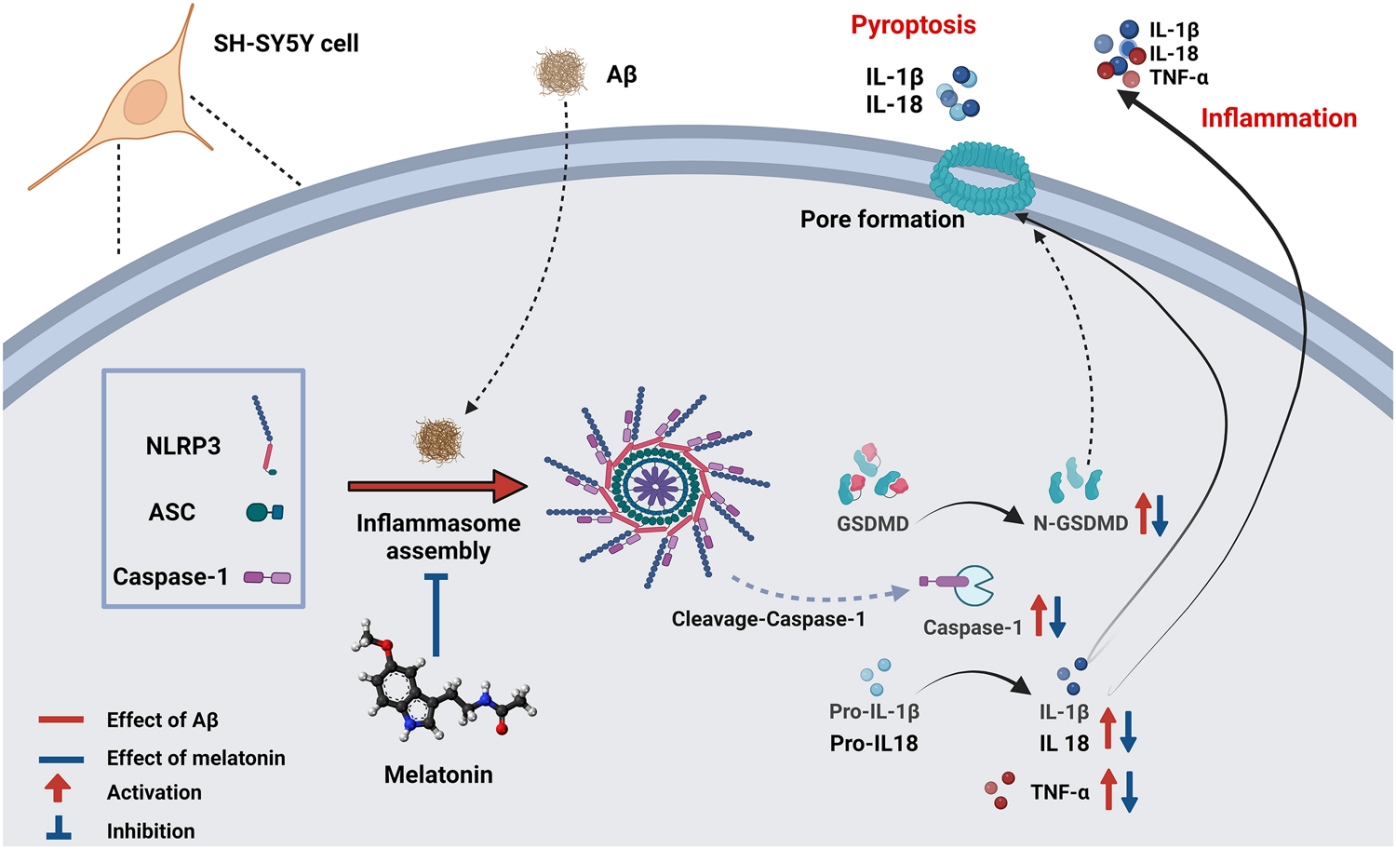
Schematic representation of the proposed mechanism by which melatonin protects against β-amyloid-mediated NLRP3 inflammasome signaling in neuronal cells leading to neuronal dysfunction. β-amyloid (Aβ) is taken up by neuronal cells and triggers NLRP3 activation and oligomerization, leading to ASC recruitment and polymerization and caspase 1 recruitment, dimerization and activation. Within the assembled inflammasome, active caspase 1 cleaves pro-IL-1β, pro-IL-18, and GSDMD into their bioactive forms: cleaved-IL-1β, cleaved -IL-18, and N-GSDMD. GSDMD-p30 forms pores in the plasma membrane and can induce cell lysis by pyroptosis. Bioactive IL-1β/IL-18 can be released through GSDMD-dependent and -independent mechanisms into the extracellular space, propagating the inflammatory signal to neighboring cells, leading to further Aβ production, creating a vicious proinflammatory cycle and perpetuating neuronal cell death. Melatonin can prevent Aβ-induced inflammasome signaling by inhibiting NLRP3, ASC and caspase 1 to induce excessive cytokine release and pyroptosis initiation by decreasing N-GSDMD levels. This figure is created with BioRender.com.

In conclusion, our research revealed that melatonin could prevent Aβ-induced inflammasome signaling by inhibiting NLRP3, ASC and caspase 1 to induce excessive cytokine release and pyroptosis by decreasing N-GSDMD levels (Fig. 8). Melatonin did not, however, affect the inflammasome through the melatonin receptor. The ability of melatonin to inhibit inflammasome activity could herald a new era in the management of inflammation-related AD progression.

### Supplementary Information

Supplementary Figures.

## Data Availability

The data used to support the findings of this study are available from the corresponding author upon request.

## Abbreviations

Aβ: Amyloid beta
AD: Alzheimer's disease
ASC: Apoptosis-associated speck-like protein containing a CARD
caspase: Cysteinyl aspartate-specific proteinases caspases
GSDMD: Gasdermin D
N-GSDMD: N-terminal gasdermin D
IL-1β: Interleukin-1β
INF-4E: Ethyl 2[(2-chlorophenyl)(hydroxyl)methyl]acrylate
NLRP3: NOD-like receptor family pyrin domain-containing 3
PRRs: Proinflammatory pattern recognition receptors
ROS: Reactive oxygen species
TNF-α: Tumor necrosis factor
