# Supplementary material for: The role of melatonin in amyloid beta-induced inflammation mediated by inflammasome signaling in neuronal cell lines
Source: Sci Rep. 2023 Oct 19;13:17841. doi: 10.1038/s41598-023-45220-1 (PMC10587142; doi:10.1038/s41598-023-45220-1)

The Role of Melatonin in Amyloid Beta-Induced Inflammation Mediated by Inflammasome Signaling in Neuronal Cell Lines

Chutikorn Nopparat[1], Anuttree Boontor[2], Suchanoot Kutpruek[2] and Piyarat Govitrapong[2, *]

[1]Innovative Learning Center, Srinakharinwirot University, Sukhumvit 23, Bangkok 10110, Thailand

[2]Chulabhorn Graduate Institute, Chulabhorn Royal Academy, Laksi, Bangkok, Thailand

Supplementary Figures.

Supplementary Figure 1. (a, b, c, d, e, f, g, h)

Fig 1. The concentration-dependent effect of Aβ on inflammasome protein and proinflammatory cytokine expression in SH-SY5Y cells. Cells were incubated with various concentration (01, 1 and 2 μM) of Aβ for 24 min. Western blot analysis was used to determine the expression levels of a) NLRP3, b) ASC, c) Caspase 1, d) pro-IL-1β, e) IL-1β, f) pro-IL-18, g) IL-18 and h) TNF-α. The band densities were normalized to actin. The ratios were calculated as a percentage of the respective value in the control group. The data are expressed as the means ± S.E.M. One-way ANOVA and Tukey's pos-hoc test were performed for statistical analysis. N=3-4 (*, **, ***, **** denote statistical significance at p< 0.05, p < 0.01, p < 0.001 and p < 0.0001 compared to the control group, respectively).

Supplementary Fig 1a. Full blot of Aβ treatment on NLRP3 expression in SH-SY5Y cells.

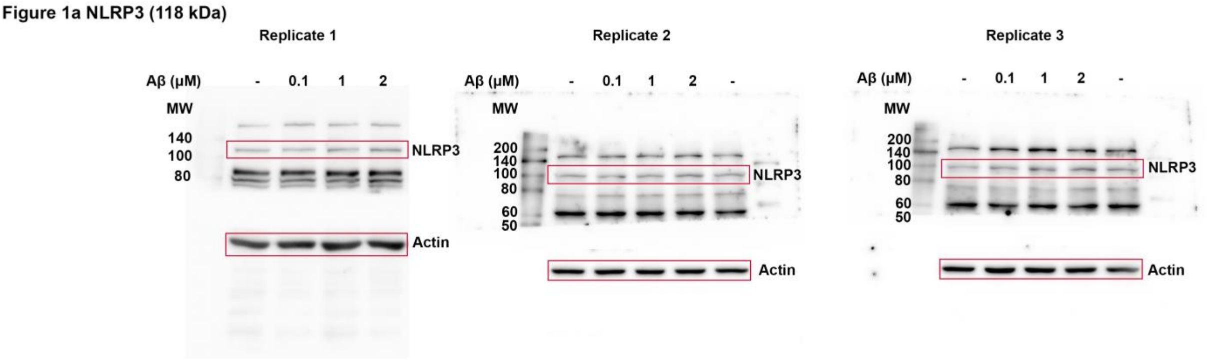

Supplementary Fig 1b. Full blot of Aβ treatment on ASC expression in SH-SY5Y cells

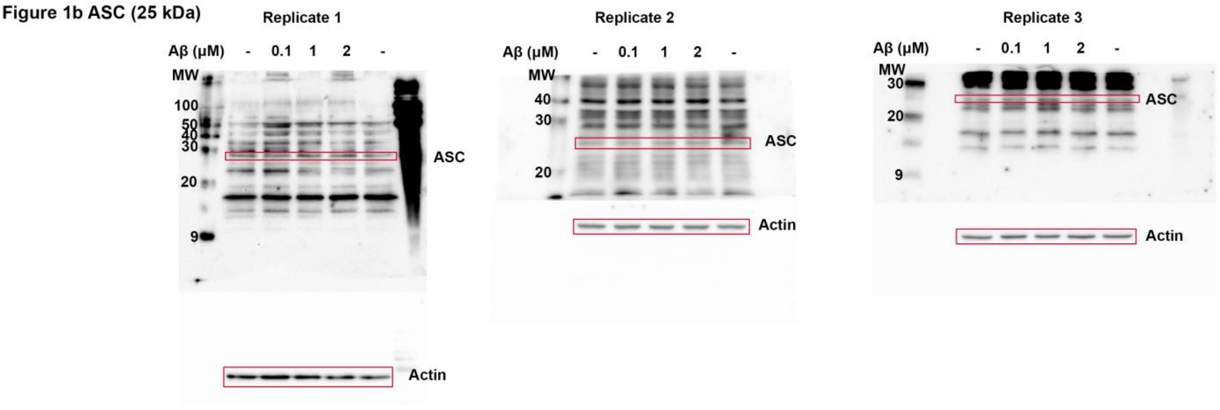

Supplementary Fig 1c. Full blot of Aβ treatment on caspase 1 expression in SH-SY5Y cells

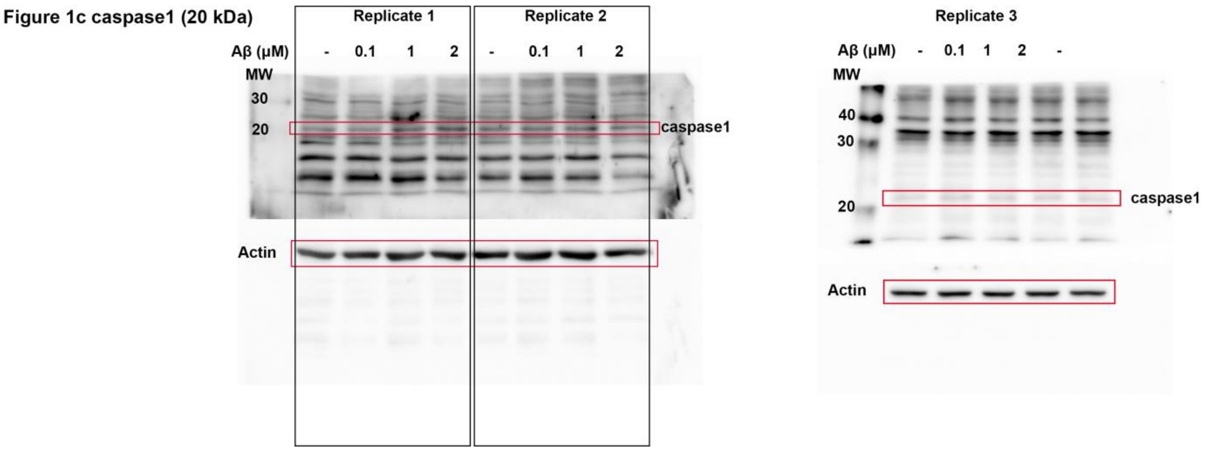

Supplementary Fig 1d. Full blot of Aβ treatment on pro-IL-1β expression in SH-SY5Y cells

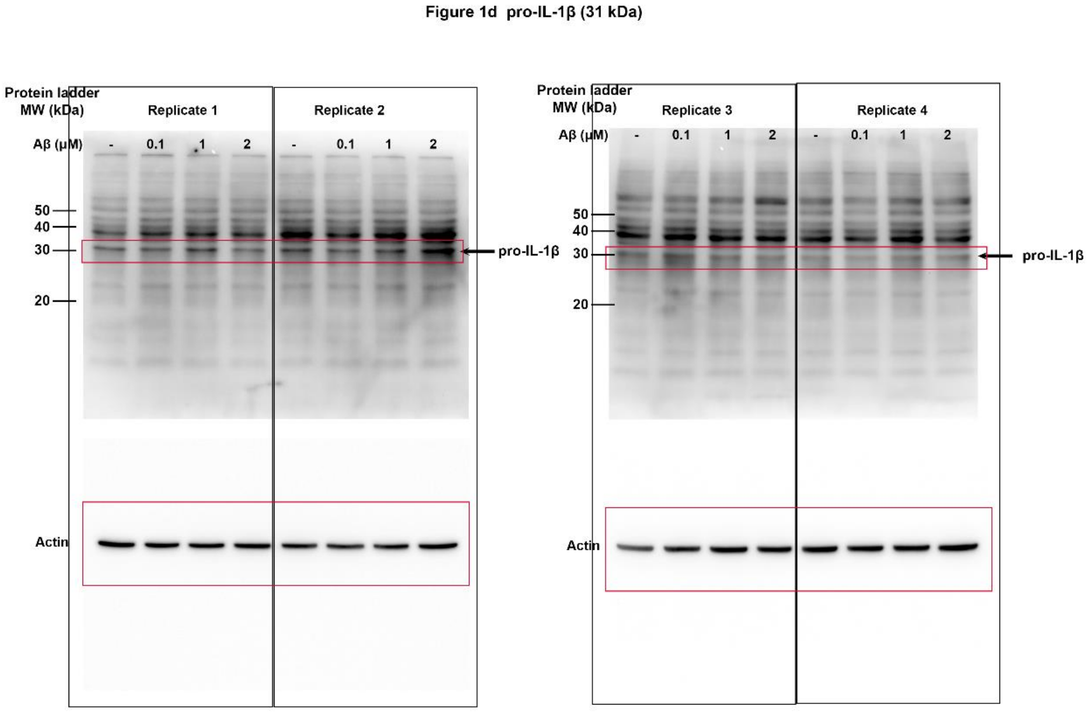

Supplementary Fig 1e. Full blot of Aβ treatment on cleaved-IL-1β expression in SH-SY5Y cells

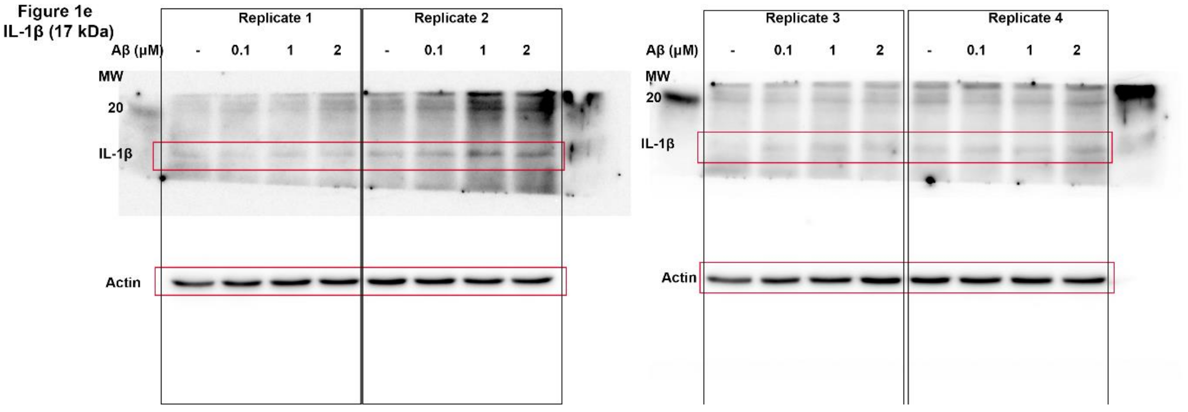

Supplementary Fig 1f and 1g. Full blot of Aβ treatment on pro- and cleaved-IL-18 expression in SH-SY5Y cells

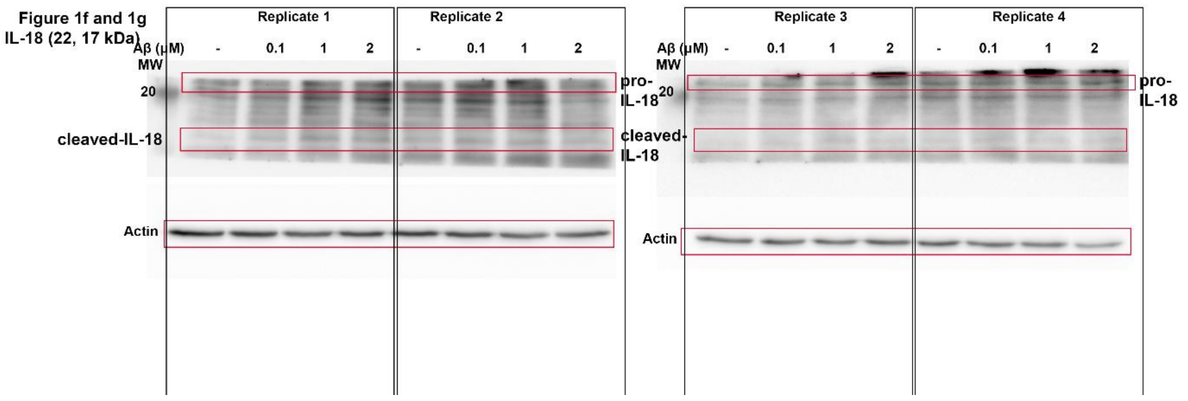

Supplementary Fig 1h. Full blot of Aβ treatment on TNF-α expression in SH-SY5Y cells

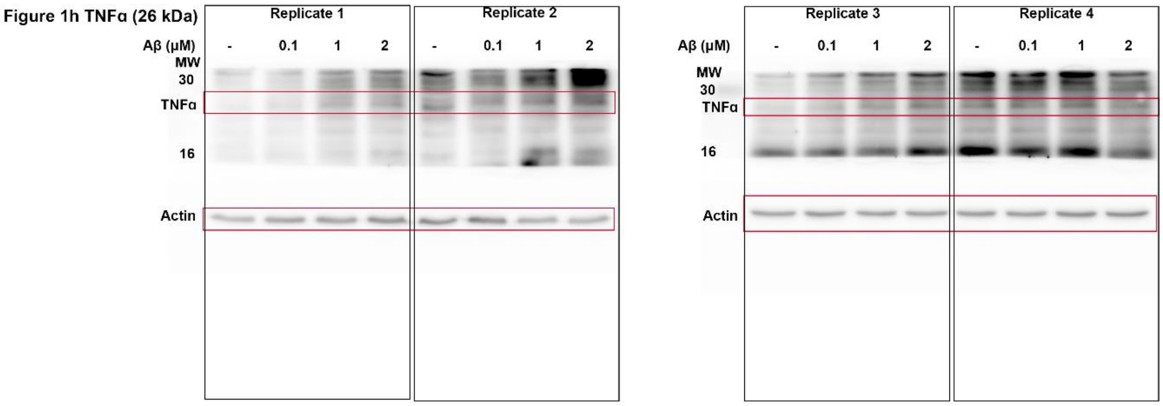

Supplementary Figure 2. (a, b, c, d)

Fig. 2. The concentration-dependent effect of melatonin on Aβ-induced inflammasome proteins expression in SH-SY5Y cells. Cells were pretreated with 1 µM or 10 µM melatonin for 2 h followed by 1 µM Aβ treatment. Western blot analysis was used to determine the expression levels of a) inflammasome proteins including NLRP3, b) ASC, c) pro-Caspase 1 and d) cleaved-caspase 1. The band densities were normalized to actin. The ratios were calculated as a percentage of the respective value in the control group. The data are expressed as the means ± S.E.M. one-way ANOVA and Tukey's pos-hoc test were performed for statistical analysis. N=3 (*, **, **** denote statistical significance at $p< 0.05$, $p < 0.01$, and $p < 0.0001$ compared to the control group, and ##, ###, #### denote statistical significance at $p < 0.01$, $p < 0.001$, and $p < 0.0001$ compared to the Aβ treatment group, respectively).

Supplementary Fig 2a. Full blot of melatonin pretreatment on NLRP3 expression in

SH-SY5Y cells

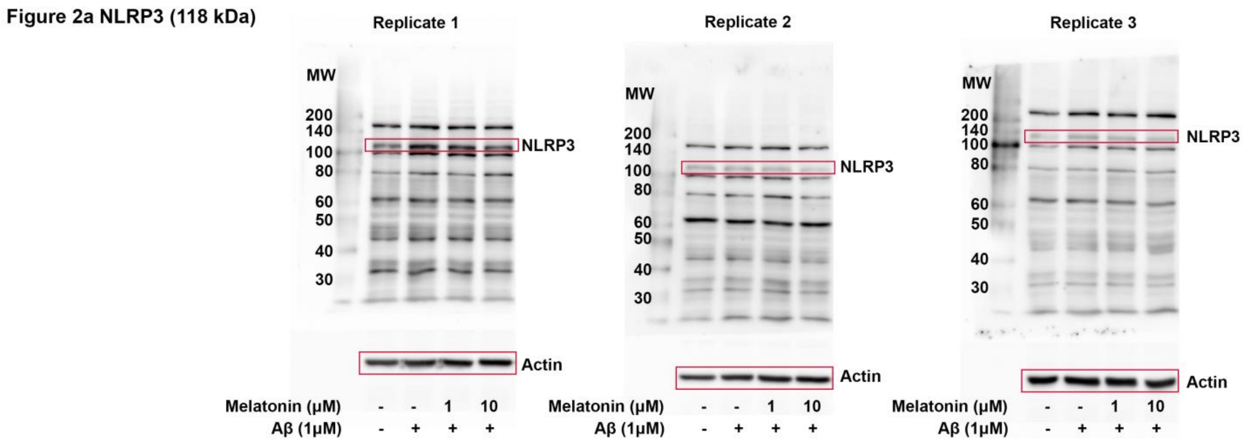

Supplementary Fig 2b. Full blot of melatonin pretreatment on ASC expression in SH-SY5Y cells

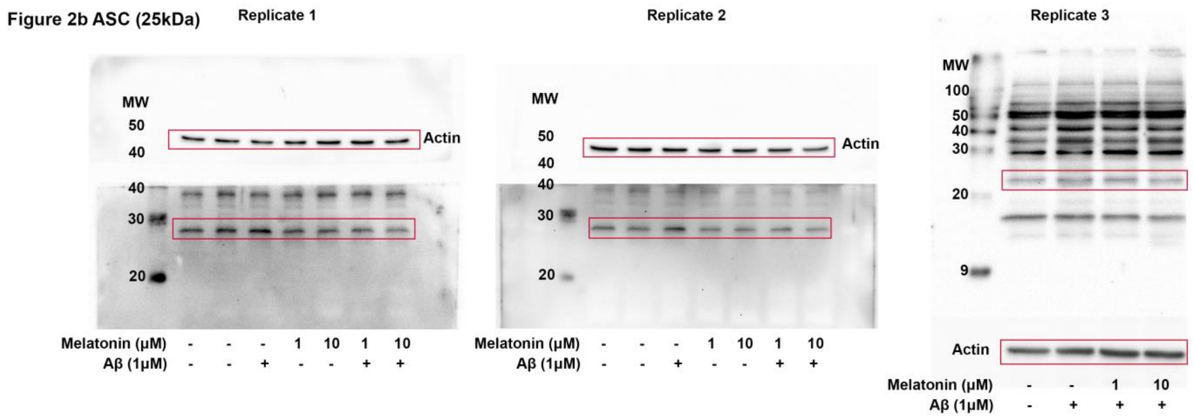

Supplementary Fig 2c, 2d. Full blot of melatonin pretreatment on pro-caspase 1 and cleaved-caspase 1 expressions in SH-SY5Y cells

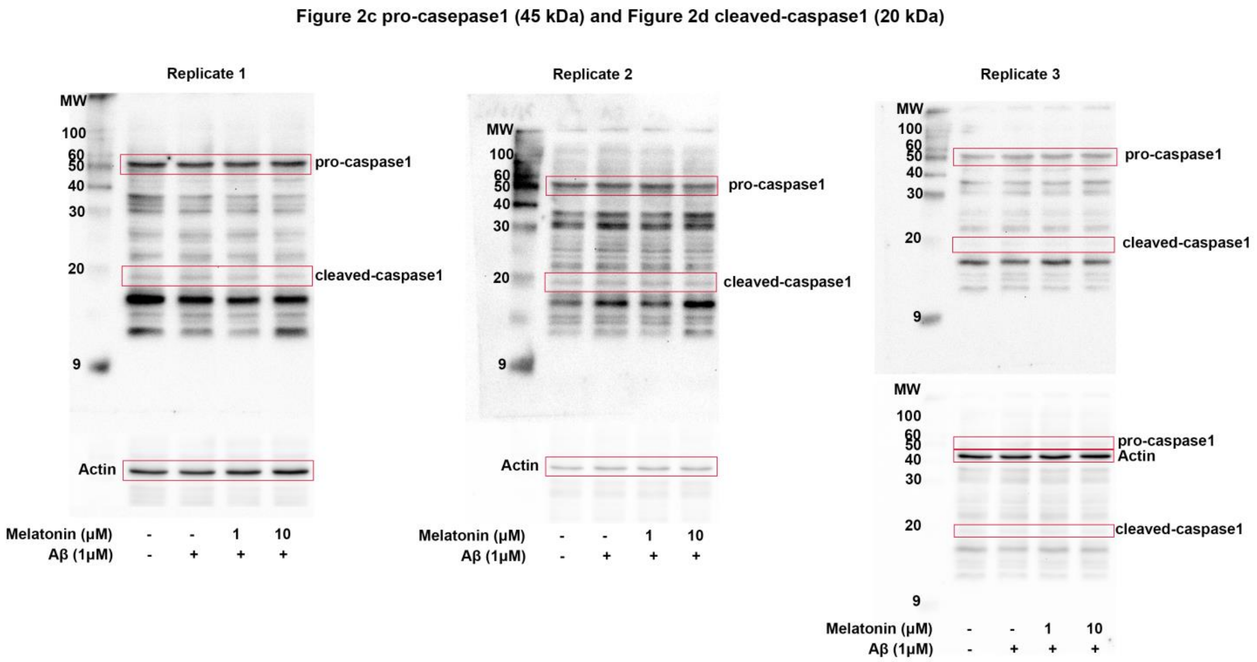

Supplementary Figure 4. (a, b, c, d, e)

Fig. 4. The concentration-dependent effect of melatonin on Aβ-induced cytokine expression in SH-SY5Y cells. Cells were pretreated with 1 μM or 10 μM melatonin for 2 h and followed by 1 μM Aβ treatment. Western blot analysis was used to determine the expression levels of cytokines including a) pro-IL-1β, b) IL-1β, c) pro-IL-18, d) IL-18 and e) TNF-α. The band densities were normalized to actin. The ratios were calculated as a percentage of the respective value in the control group. The data are expressed as the means ± S.E.M. One-way ANOVA and Tukey's pos-hoc test were performed for statistical analysis. N=3 (*, ****denote statistical significance at $p < 0.05$ and 0.0001 compared to the control group, and #, ## denote statistical significance at $p < 0.05$ and $p < 0.01$, compared to the Aβ treatment group, respectively).

Supplementary Fig 4a and b. Full blot of melatonin pretreatment on IL-1β expression in SH-SY5Y cells

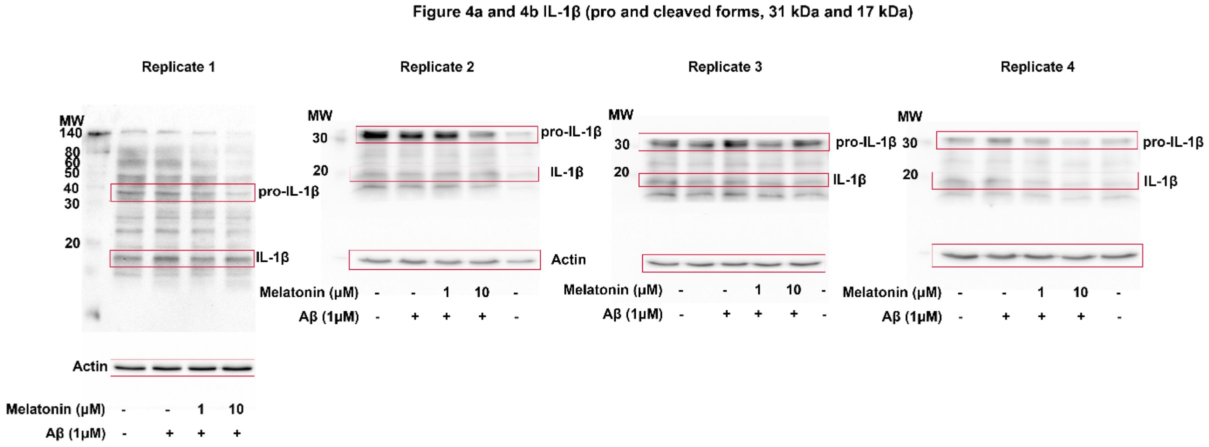

Supplementary Fig 4c and d. Full blot of melatonin pretreatment on IL-18 expression in SH-SY5Y cells

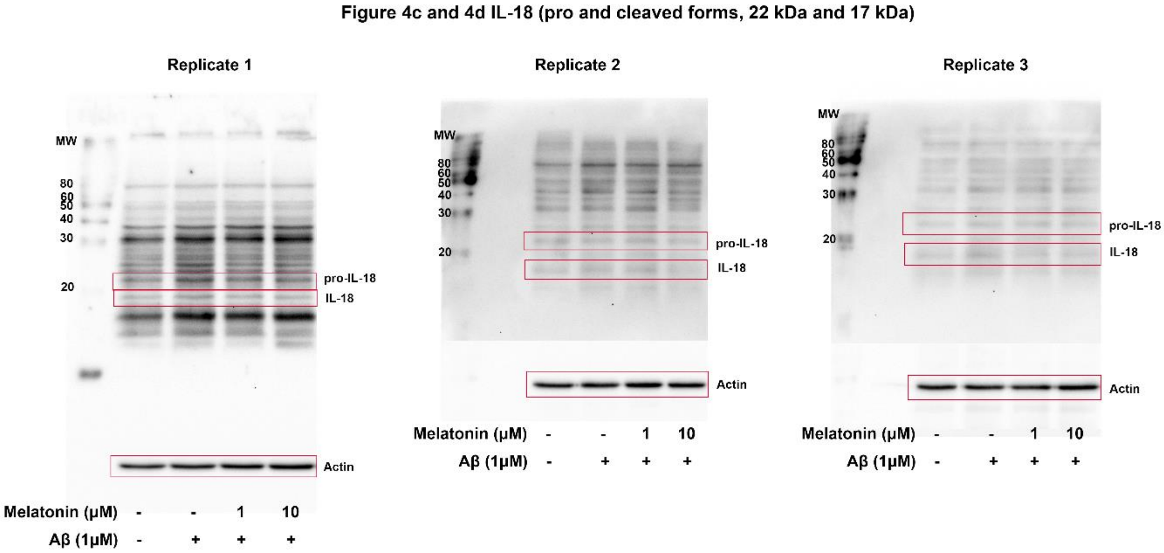

Supplementary Fig 4e. Full blot of melatonin pretreatment on TNF-α expression in SH-SY5Y cells

Figure 4e TNFα (26 kDa)

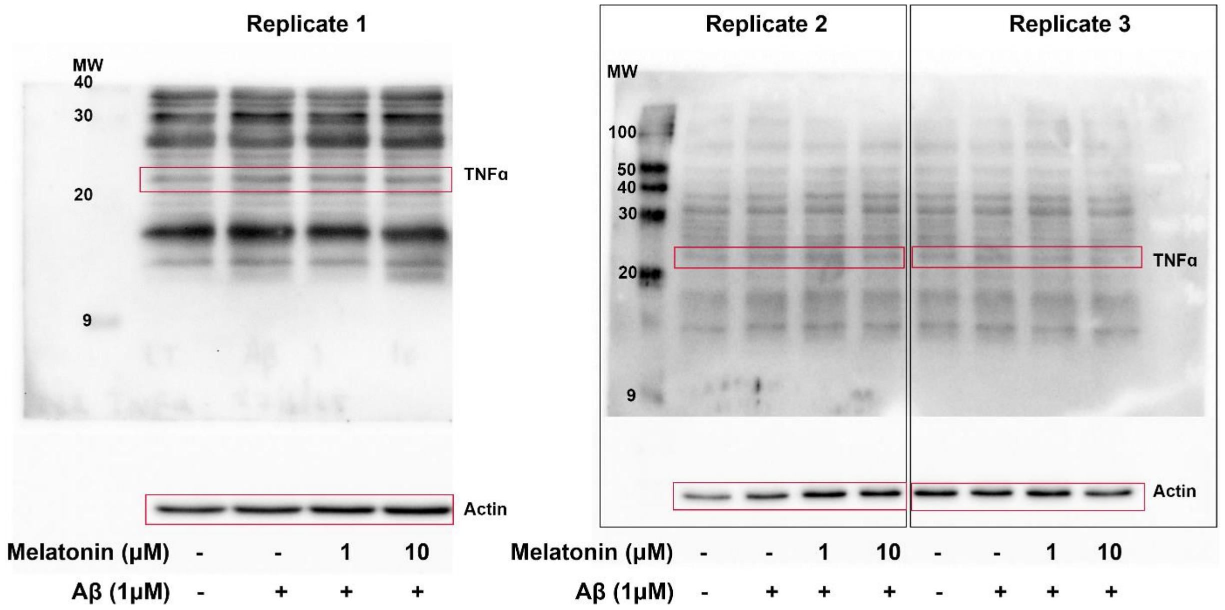

Supplementary Figure 6. (a, b, c, d, e)

Fig. 6. Protective effects of melatonin against Aβ-induced cytokines via the melatonin receptor in SH-SY5Y cells. Cells were treated with 1 µM luzindole 30 min prior to pretreatment with 10 µM melatonin for 2 h followed by 1 µM Aβ treatment. Western blot analysis was used to determine the expression levels of inflammatory cytokines including a) pro-IL-1β, b) IL-1β, c) pro-IL-18, d) IL-18 and e) TNF-α. The band densities were normalized to actin. The ratios were calculated as a percentage of the respective value in the control group. The data are expressed as the means ± S.E.M. One-way ANOVA and Tukey's pos-hoc test were performed for statistical analysis. N=4 (*, **** denote statistical significance at $p< 0.05$, $p < 0.0001$ compared to the control group; ##, ### denote statistical significance at $p < 0.01$ and $p < 0.001$ compared to the Aβ treatment group, respectively; and *f* and ff denote statistical significance at $p < 0.05$, 0.01 compared to melatonin pretreatment group).

Supplementary Fig 6a and b. Full blot of luzindole on IL-1β expression in SH-SY5Y cells

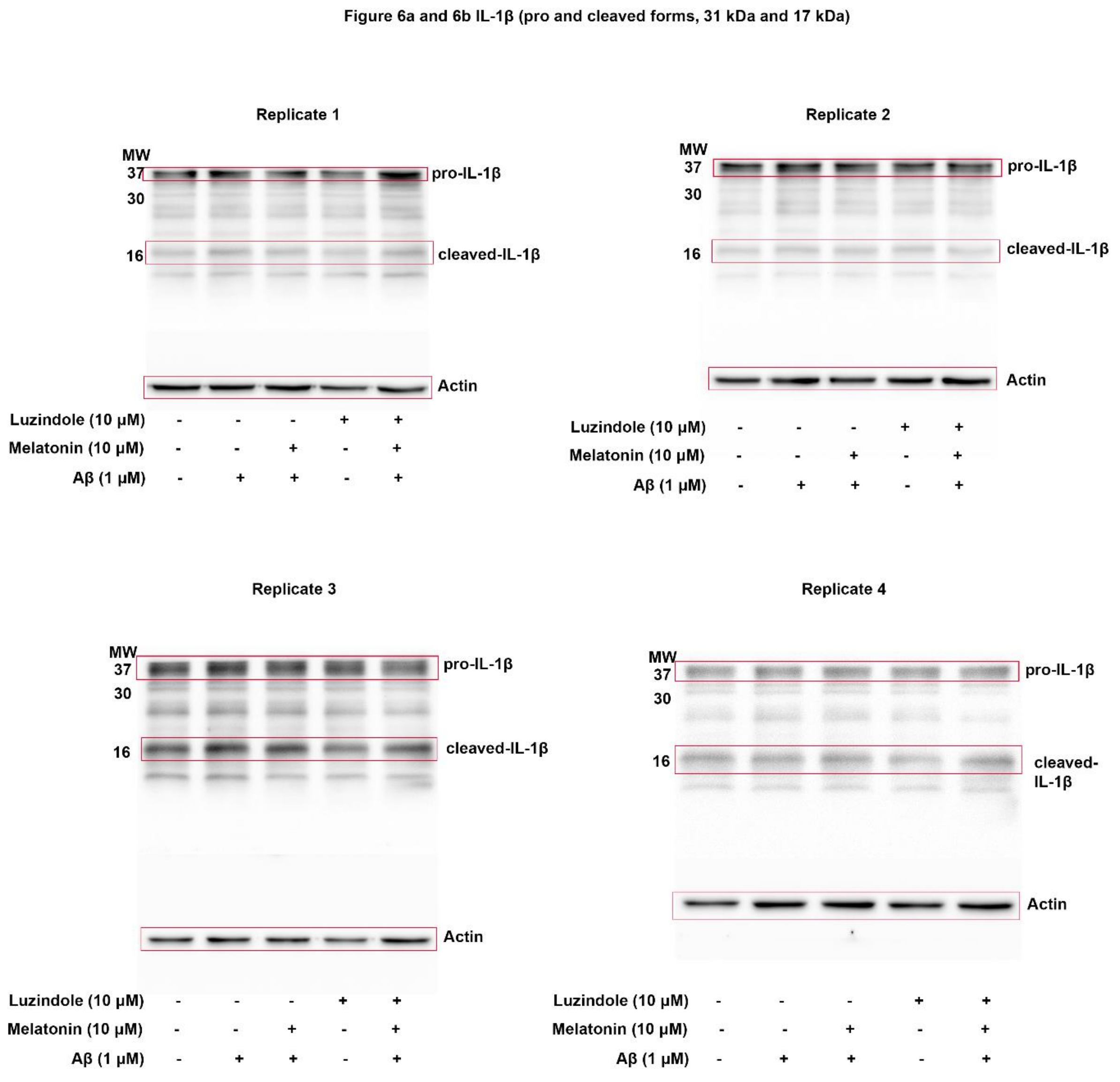

Supplementary Fig 6c and d. Full blot of luzindole on IL-18 expression in SH-SY5Y cells

Figure 6c and 6d IL-18 (pro and cleaved forms, 22 kDa and 17 kDa)

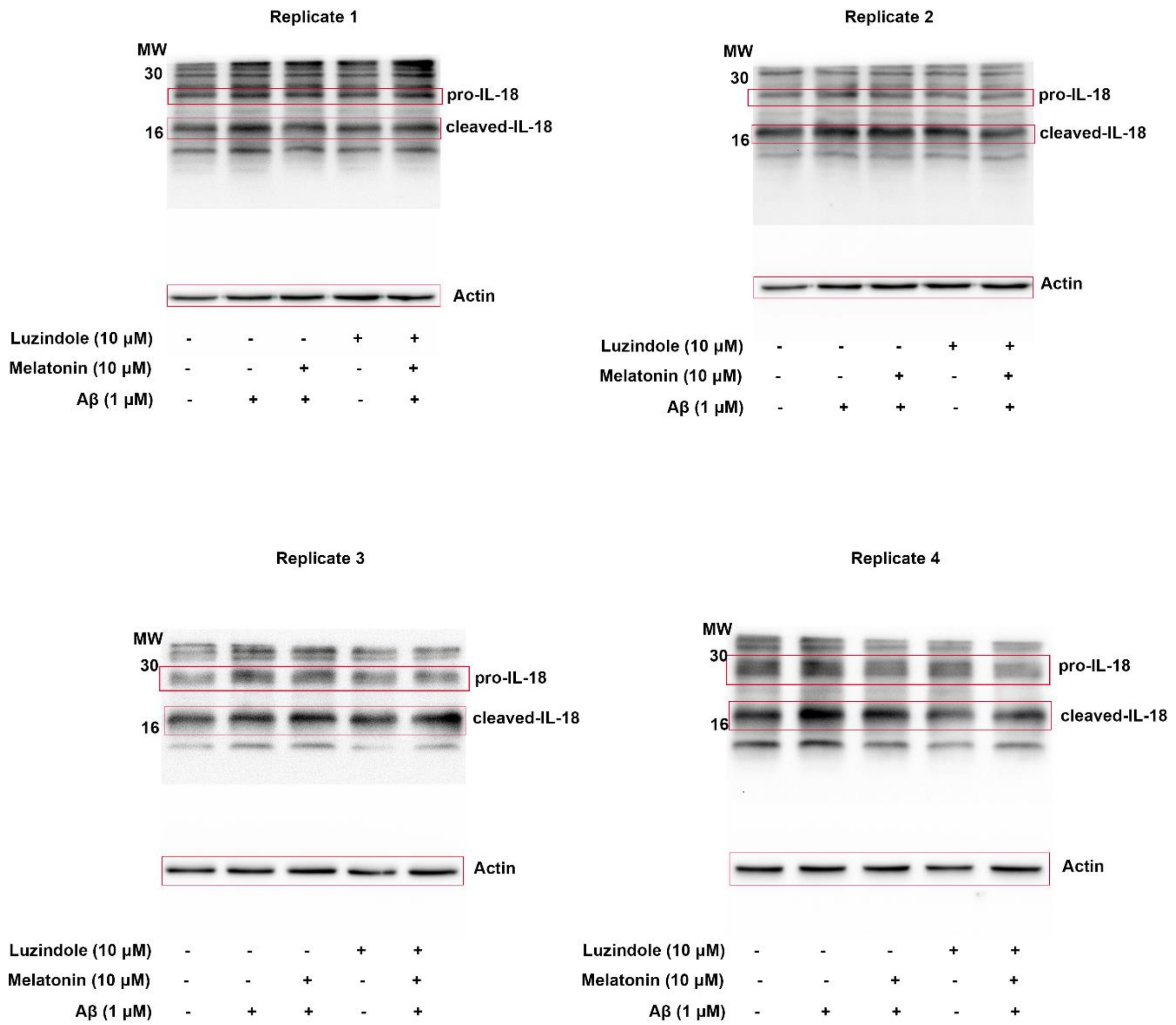

Supplementary Fig 6e. Full blot of luzindole on TNF-α expression in SH-SY5Y cells

Figure 6e TNFα (26 kDa)

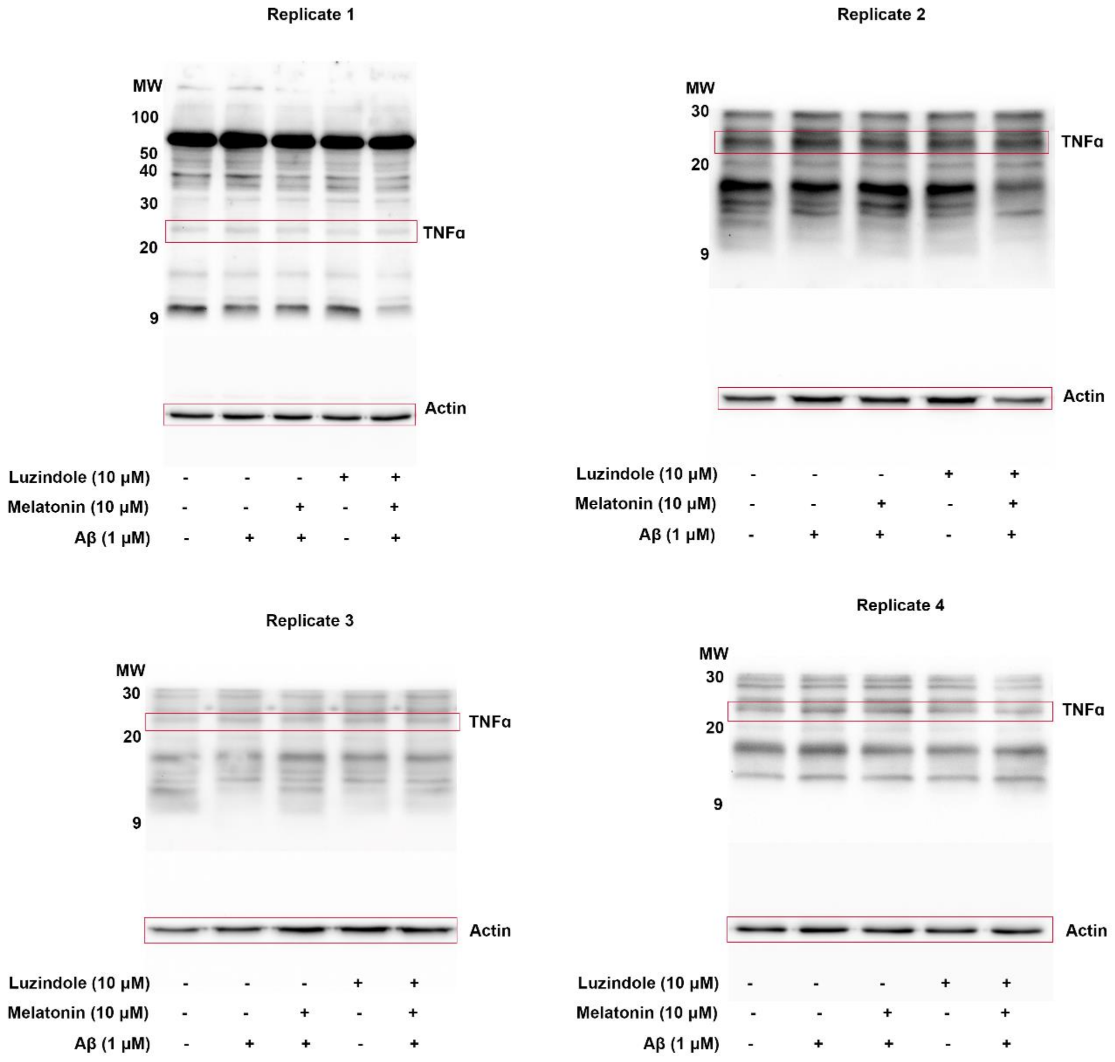

Supplementary Figure 7. (a, b, c, d, e, f, g, h)

Fig. 7. Effects of cytokines release via the inflammasome in Aβ-treated SH-SY5Y cell. Cells were treated with 1 µM INF-4E (NLRP3, caspase 1 inhibitor) 30 min prior to pretreatment with 10 µM melatonin for 2 h followed by 1 µM Aβ treatment. Western blot analysis was used to determine the expression levels of a) pro-IL-1β, b) cleaved-IL-1β, c) cleaved/pro-IL-1β, d) pro-IL-18, e) cleaved-IL-18, f) cleaved/pro-IL-18 and g) TNF-α and h) Gasdermin D. The ratios were calculated as a percentage of the respective value in the control group. The data are expressed as the means ± S.E.M. One-way ANOVA and Tukey's pos-hoc test were performed for statistical analysis. N=4 (*, **, **** denote statistical significance at $p < 0.05$, $p < 0.01$, and $p < 0.0001$ compared to the control group and #, ##, ### denote statistical significance at $p < 0.05$, $p < 0.01$ and $p < 0.001$ compared to the Aβ treatment group, respectively).

Supplementary Fig 7a, 7b. Full blot of INF-4E on pro-IL-1β and cleaved-IL-1β expressions in SH-SY5Y cells

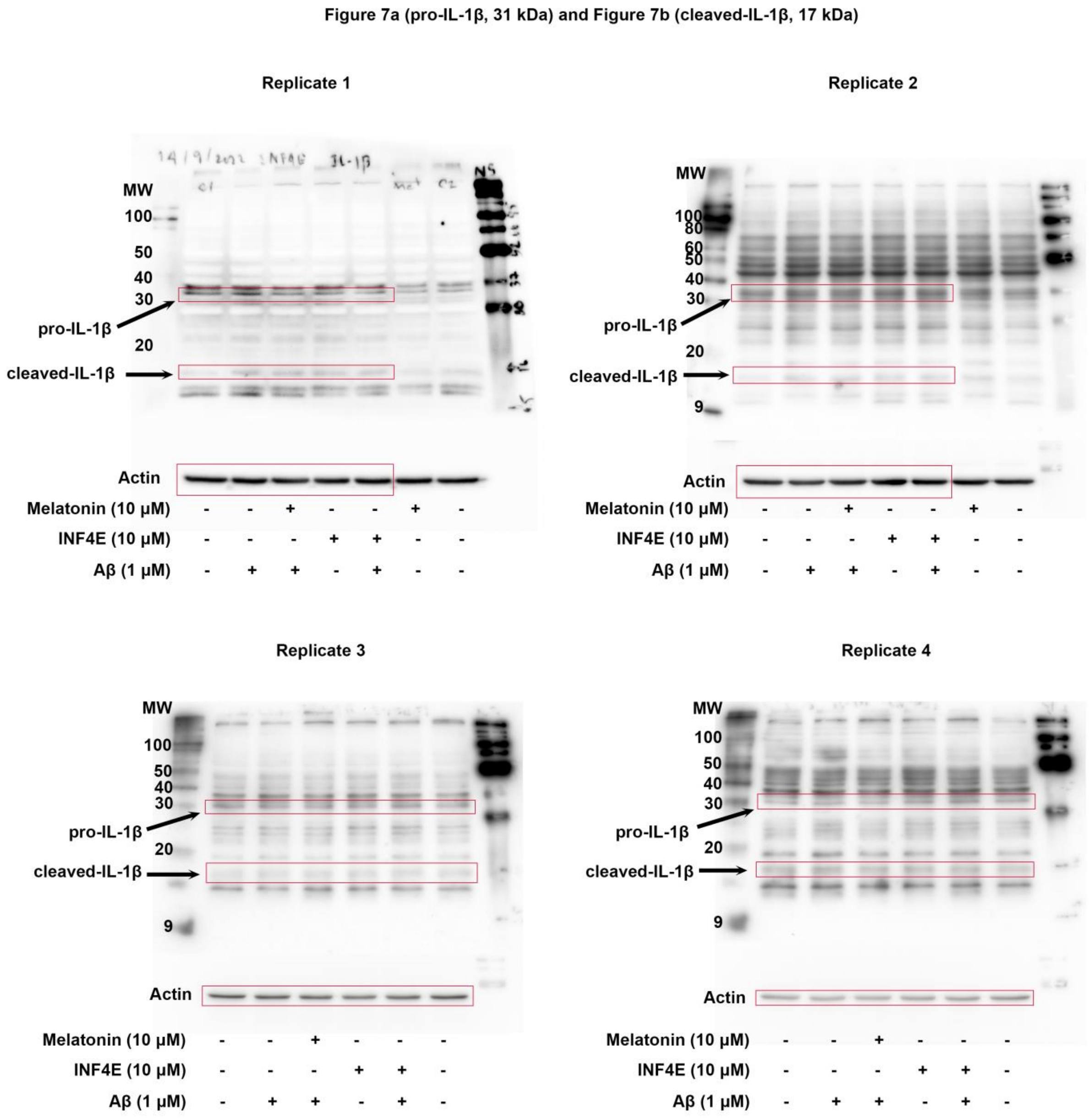

Supplementary Fig 7d, 7e. Full blot of INF-4E on pro-IL-18 and cleaved-IL-18 expressions in SH-SY5Y cells

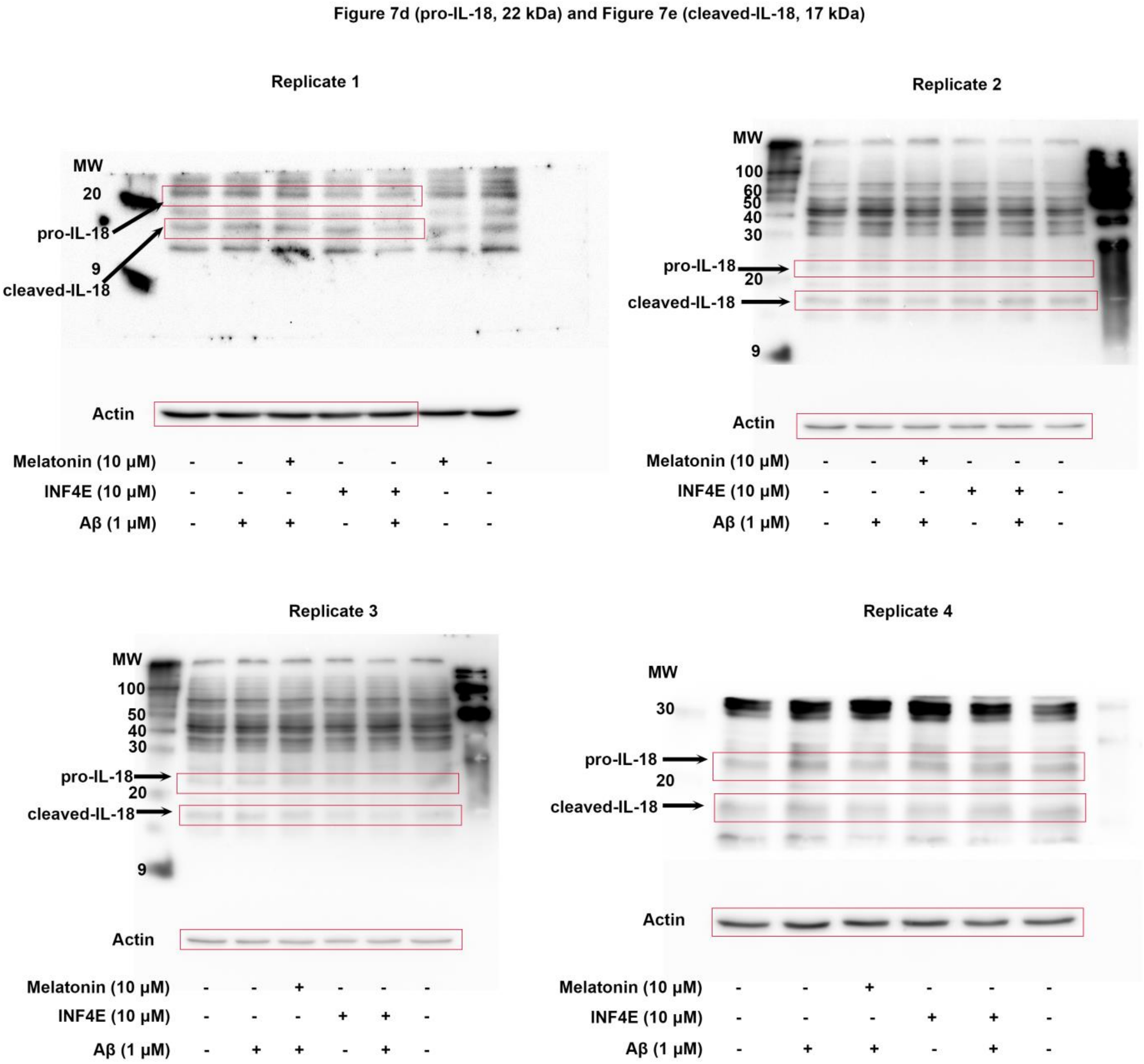

Supplementary Fig 7g. Full blot of INF-4E on TNF-α expression in SH-SY5Y cells

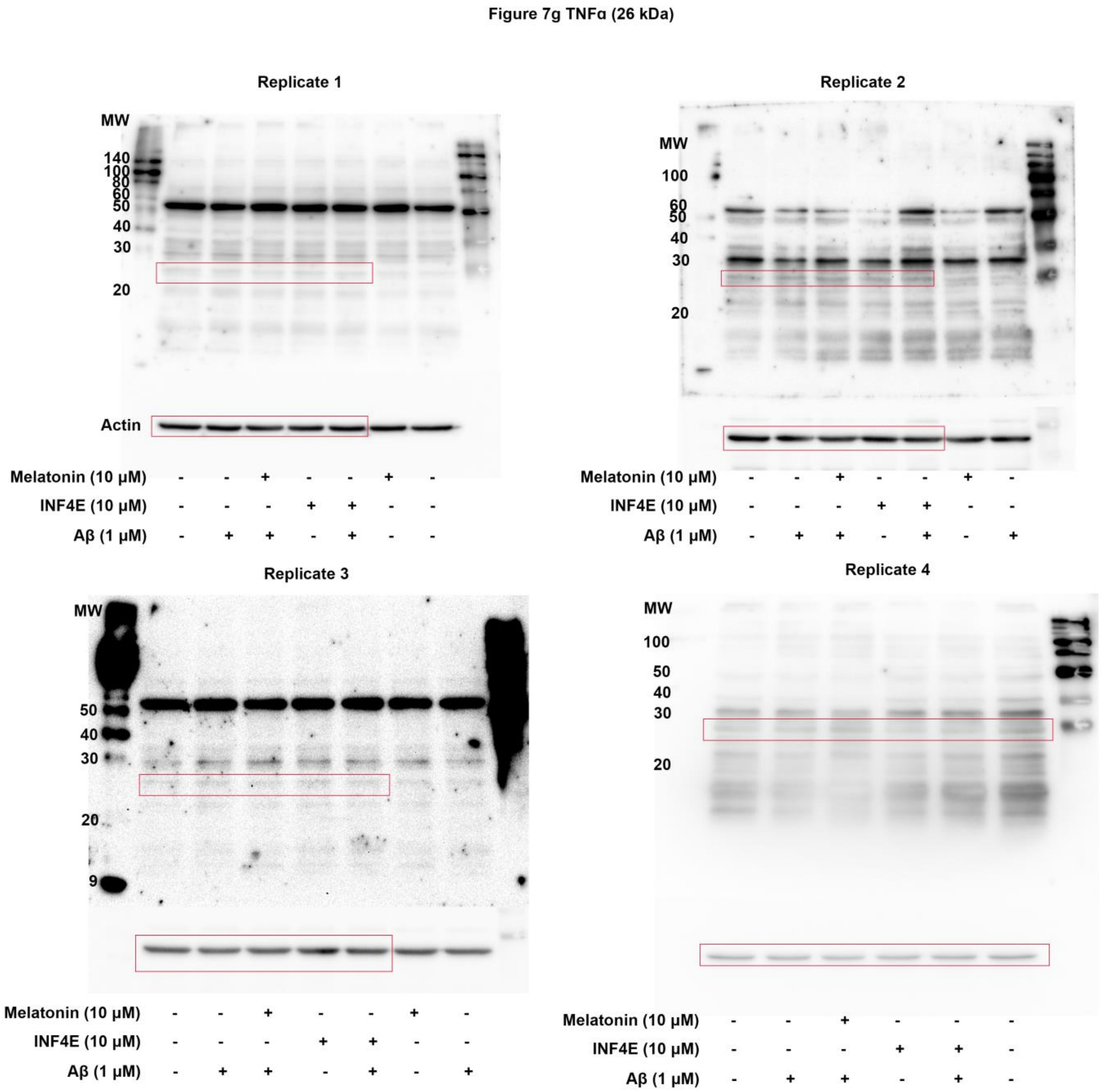

Supplementary Fig 7h. Full blot of INF-4E on N-GSDMD expression in SH-SY5Y cells

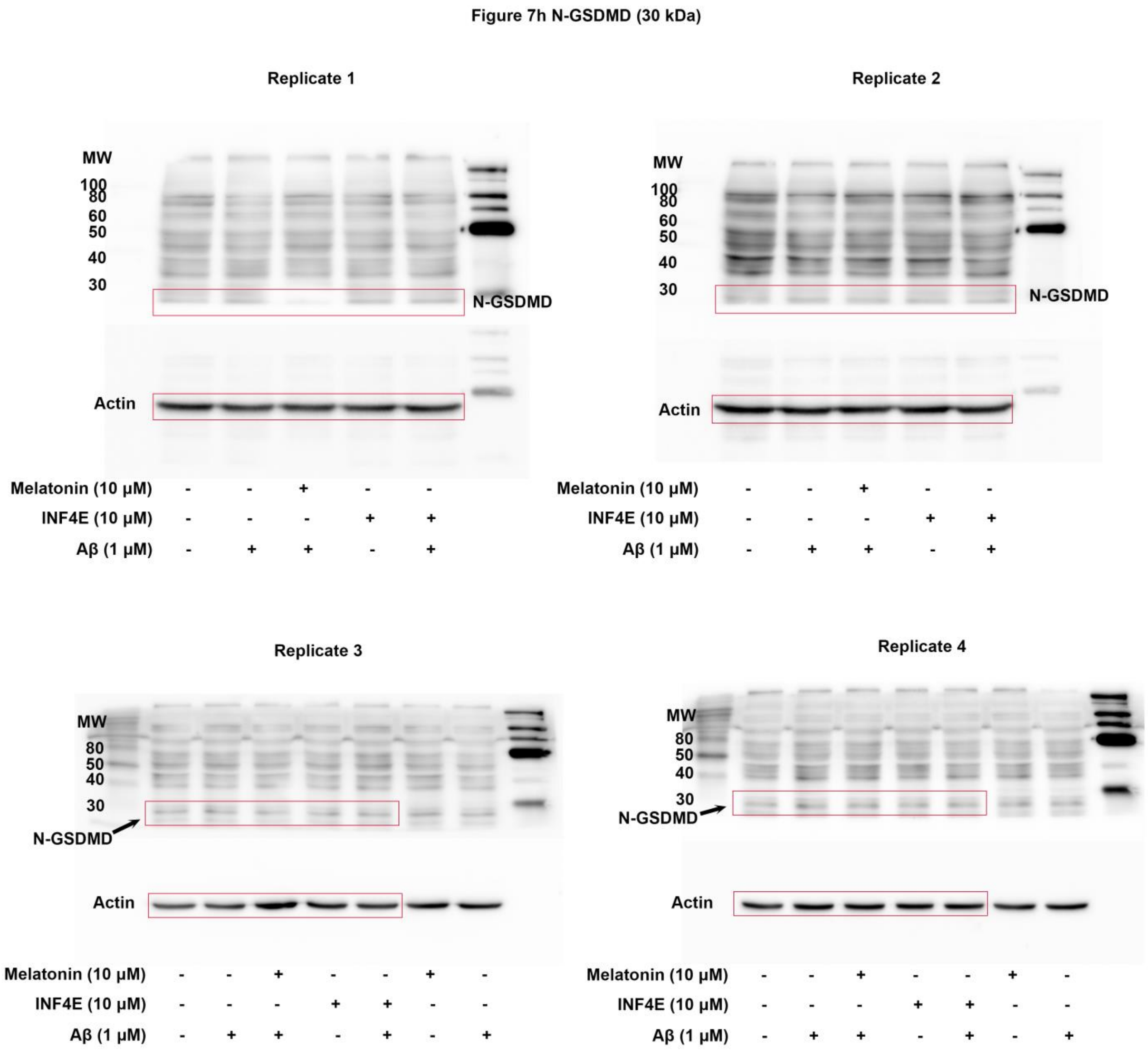

Supplement: Supplementary file 1 — Supplementary Figures. [file 41598_2023_45220_MOESM1_ESM.pdf]
